# Establishing an inexpensive, space efficient colony of *Bemisia tabaci* MEAM1 utilizing modelling and feedback control principles

**DOI:** 10.1111/jen.12995

**Published:** 2022-03-24

**Authors:** Natalie M. Thompson, Nadia Waterton, Antonios Armaou, Jane E. Polston, Wayne R. Curtis

**Affiliations:** ^1^ 8082 Department of Chemical Engineering The Pennsylvania State University University Park Pennsylvania USA; ^2^ 8082 Department of Mechanical Engineering The Pennsylvania State University University Park Pennsylvania USA; ^3^ Department of Plant Pathology University of Florida Gainesville Florida USA

**Keywords:** cost reduction, crop protection, image analysis, insectary, proliferation kinetics, quality control, whitefly

## Abstract

A stable, synchronized colony of whitefly (*Bemisia tabaci* MEAM1 Gennadius) was established in a single ~30 cu.ft. reach‐in incubator and supported on cabbage host plants which were grown in a 2 × 2′ mesh cage without the need for a greenhouse or dedicated growth rooms. The colony maintenance, including cage cleaning and rotation of plants, was reduced to less than 10 h per week and executed by minimally experienced researchers. In our hands, this method was approximately 10‐fold less expensive in personnel and materials than current typical implementations. A predator‐prey model of whitefly colony maintenance that included whitefly proliferation and host plant health was developed to better understand and avoid colony collapse. This quantitative model can be applied to inform decisions such as inoculum planning and is a mathematical framework to assess insect control strategies. Extensive measurements of colony input and output (such as image analysis of leaf area and whitefly population size) were performed to define basic ‘feedback control’ parameters to gain reproducibility of this inherently unstable scaled‐down whitefly colony. Quantitative transfer of ~100 whiteflies repeatedly produced more than 5000 adult whiteflies over a 6‐week, two‐generation period. Larger scale experimentation could be easily accommodated by transferring adult whiteflies from the maintenance colony with a low flow vacuum capture device. This approach to colony maintenance would be useful to programs that lack extensive plant growth room or greenhouse access and require a “clean” implementation that will not contaminate an axenic tissue culture laboratory.

## 1 INTRODUCTION

Whiteflies (*Bemisia tabaci* MEAM1 Gennadius) are phloem‐feeding insects that are responsible for substantial crop losses due in large part to the viruses that they transmit. Among the viruses that they transmit is the tomato yellow leaf curl virus (TYLCV), which is well known among the agricultural community due to its prevalence and damage (Czosnek et al., 2001; Hunter et al., 2007; Zeidan & Czosnek, 1991). Researching whiteflies and their transmitted viruses is an important aspect of developing a plan to mitigate crop loss.

One of the barriers to carrying out research on whitefly‐transmitted pathogens is the required establishment and maintenance of a whitefly colony and the considerable cost of operation in personnel, materials and facilities. Typically studies of whitefly virus transmission require isolation of viruliferous and virus‐free colonies using separate rooms or greenhouses, as well as the use of small greenhouses to produce host plants for the colonies (Lapidot et al., 2014; Polston & Capobianco, 2013; Schuster et al., 2009). An additional obstacle to whitefly‐vector research is that many of the current methods utilized are a potential point of contamination – a particularly prevalent concern is the routine introduction of host plants usually produced in a greenhouse. While some of these limitations can be overcome through collaborations, such multi‐investigator projects are limited due to the size of grant necessary to execute the research as well as problems with meeting phytosanitary regulations. More importantly, the fragile nature of whitefly as a research component makes it time‐consuming and difficult to transport between performance locations. We designed, implemented and refined a scaled‐down version of a whitefly colony that avoids the aforementioned obstacles by being readily implemented in an indoor laboratory at minimal cost with an accompanied model to predict the quantity of whiteflies available for experimentation.

Whiteflies have a roughly 3‐week life cycle for feeding, egg‐lay and nymph development at 28°C. At this temperature, whitefly females are capable of laying 50–110 eggs (Aregbesola et al., 2020; Butler et al., 1983; Powell & Bellows, 2009), resulting in an amplification ratio that can cause a rapid decline in host plants, which is particularly problematic for scaled‐down colony maintenance (see Supplemental Figure S8‐1). Given the haplodiploidy nature of whiteflies, unfertilized eggs will hatch as male; therefore, a colony initiated from small numbers of whiteflies can result in a male/female imbalance. The method developed here is our ‘engineering’ solution to this challenge of reducing cost and effort while still maintaining robust and high‐quality whiteflies for research studies.

## 2 MATERIALS AND METHODS

### 2.1 Indoor facilities design

The colony was maintained in a single reach‐in incubator (SP Scientific, model 317512, 33 cu.ft. Environmental Stability Chamber) that had sufficient inner dimensions (33″W × 27″D × 60″H) to accommodate four cages that would provide two whitefly life cycles. Cages were designed to accommodate front‐loaded standard 1020 greenhouse flat trays (Agron #HGC726165) and whiteflies using whitefly‐proof screening. The optimal design was 12″W × 24″L × 18″H with a 12″ × 12″ front panel door reach‐in sock with a 6″ vinyl upper segment window for observation of watering and plant health (BioQUIP #1450NS85). The use of a remote‐control watering pump cart made for extremely convenient and time‐efficient watering that could be systematized based on the timing of the watering per pot (see Supplemental Info S1). Lighting was provided by (6) slim line LED fixtures (Beamswork DA FDSPEC LED, 30″ with Timer module) where the aquarium attachment design facilitates three panels per level into the existing shelving brackets of the incubator.

### 2.2 Cabbage host plants

Cabbage plants were selected as the whitefly host due to their immunity to TYLCV, ability to support high whitefly populations, relatively large leaf areas, low height and thick leaves, which helped plants resist collapse. ‘Earliana’ (W. Atlee Burpee & Co.) was selected among several cabbage cultivars due to its relatively compact growth habit and shorter time to harvest; although ‘All Seasons’ appeared to perform comparably under the warm 27°C growth conditions, its more crinkled leaves were found less amenable to surface area image analysis. Other cabbage and broccoli varieties were used and can be expected to provide comparable results. Seeds were started bi‐weekly in 5″ tall by 3.5″ wide square pots (T.O. Plastics, SVD‐355‐NP) to allow up to 18 pots per 1020 greenhouse flat, where the additional soil was observed to significantly improve performance of the plants due to the greater water retention over the initially utilized 3.5″ tall pots. Host plants were generated in a 24″W × 24″L × 24″H screened cage, where we chose to have a half visible window (BioQUIP #1450NS78). This cage was on an ambient laboratory rack under several different lighting configurations (see Supplemental Info S2 for lighting details). Daily watering of the host cabbage seedling pipeline was implemented, starting with sparse watering at the time of seeding and then weekly sub‐irrigation, which included Gnatrol to control fungus gnats (Valent, Gnatrol WDG, *Bacillus thuringiensis*, subsp. israelensis, strain AM 65–52 fermentation solids) was applied by a 3–5 min placement in a 1020 flat containing 1/2 gallon of freshly prepared Gnatrol at 1/8‐teaspoon tsp powder per gallon. Over‐watering caused visible stress; therefore, pot weight was monitored as a feedback parameter for colony maintenance. Weekly watering with a dilute fertilizer solution (1/2‐teaspoon/gallon, Peters 20‐20‐20) was applied with the intent of preventing nutrient limitation to growth.

### 2.3 Primary cage setup

Five 5‐week‐old cabbage plants were weighed as an indicator of the soil moisture content entering the whitefly colony followed by addition of up to 30 ml of water if weight was under 300 g. A picture taken from above the plants provides a basis for assessing leaf surface area at this initial time point (see Figure S7‐1). These inputs (plant size and soil moisture content) were chosen based on experience of over a year of colony maintenance as important measures of colony health. Cage initiation was implemented bi‐weekly on Monday with a goal of providing procedural consistency for synchronization of adult emergence.

### 2.4 Inoculation

**FIGURE 1:**
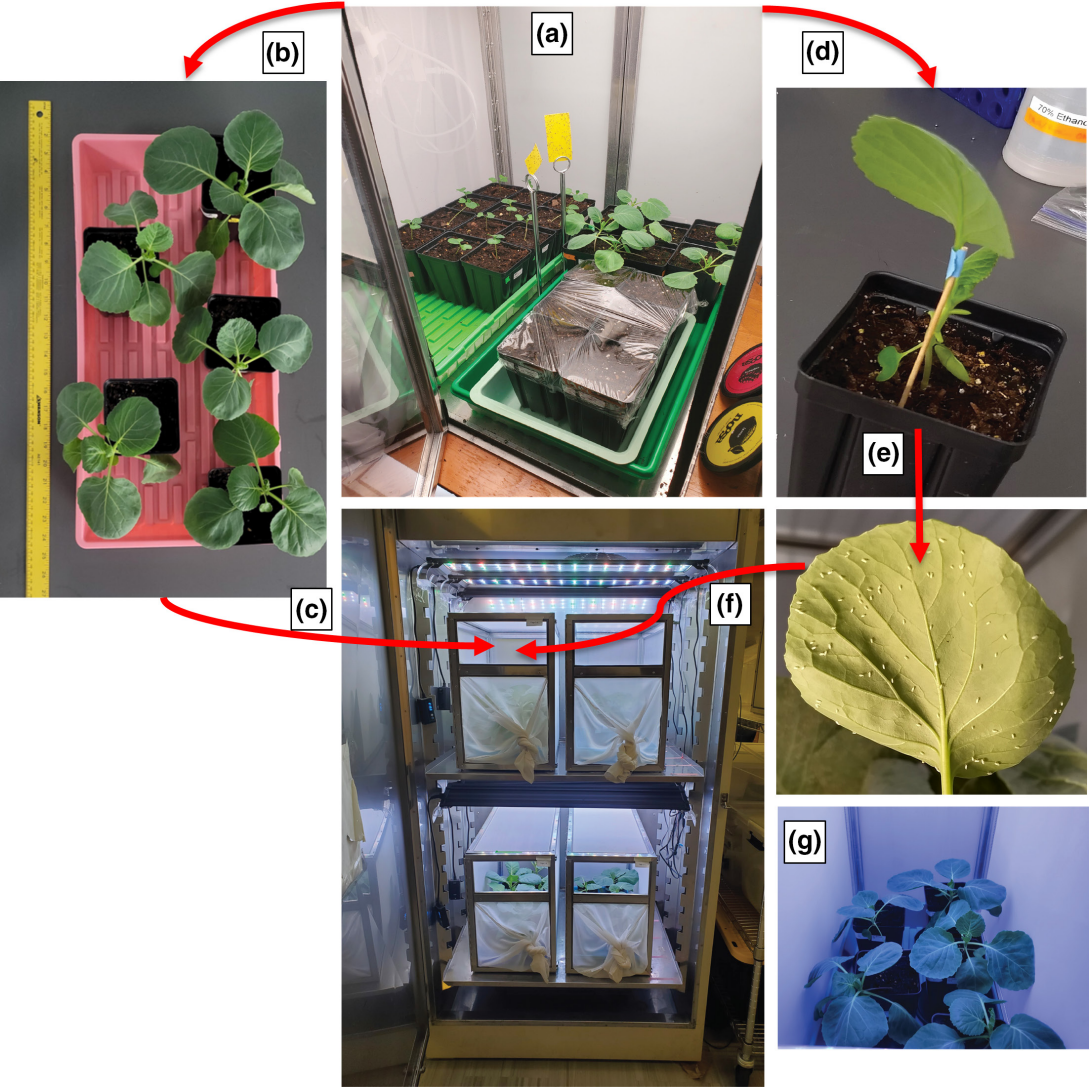
Whitefly colony maintenance configuration and methodology. (a) Cabbage host plant growth cage where 6 seedlings per cycle are grown for 5 weeks; (b) setup for digitization of the five healthiest; (c) whitefly colony incubator containing 8 weeks of bi‐weekly inoculated cages; (d) cabbage plant prepared for inoculation by leaf removal and propped for observation and photography (e) photography for quantitative whitefly counting after observation of accumulation of 80–110 whiteflies, leaf is excised into a Petri dish and (f) transferred to the new cage; (g) A picture of inoculated plants inside the colony cage [Colour figure can be viewed at wileyonlinelibrary.com]

Serial inoculation of whiteflies onto host plants underwent considerable changes in an effort to achieve reproducible colony performance. Two basic methods were found to provide an near zero‐escape of whiteflies: (1) qualitative inoculation on a transfer plant exposed for a defined period of time and (2) quantitative inoculation with a population size determined from pictures on a single leaf. Both qualitative and quantitative methods were accomplished by exposure of an inoculum plant to the colony cage entering its 7th week of proliferation. This corresponds to the emergence of the second generation of whiteflies. Qualitative inoculation was accomplished using a randomly selected host plant in an enclosed transfer device (see Figure S4‐2). Since the emphasis of this description is on quantitative colony monitoring, the details of the qualitative approach are provided in Supplemental Info S4 as that approach would be sufficient for most experimental work. For quantitative inoculation, the best approach was found to use a 5‐week‐old cabbage seedling removing all but one leaf that was fixed upright using a wooden applicator stick (see Figure 1d). The plant was illuminated obliquely with a spotlight to provide high contrast for pictures of both sides of the leaf for counting. Once the target quantity of adult whiteflies was reached, a 90 mm disposable Petri dish was used to pinch off the leaf inside the mesh cage for transfer. Wiping the outside of the Petri dish with an anti‐static dryer sheet prior to use was found to prevent whiteflies sticking to the plastic due to static charges. Inoculation involved opening the Petri dish in the newly prepared primary cage setup. Combining experience with preliminary models of whitefly proliferation, a whitefly count of 80–110 whiteflies was chosen as a balance of numbers, plant health and subsequent proliferation of both the plant host and the whiteflies.

### 2.5 Quality control parameters

The quality control (QC) procedures described below were an important aspect of establishing consistency between many different inexperienced researchers, and while not necessarily needed for routine maintenance, they are recommended for transition to new personnel during training.

#### 2.5.1 Whitefly sticky card count

**FIGURE 2:**
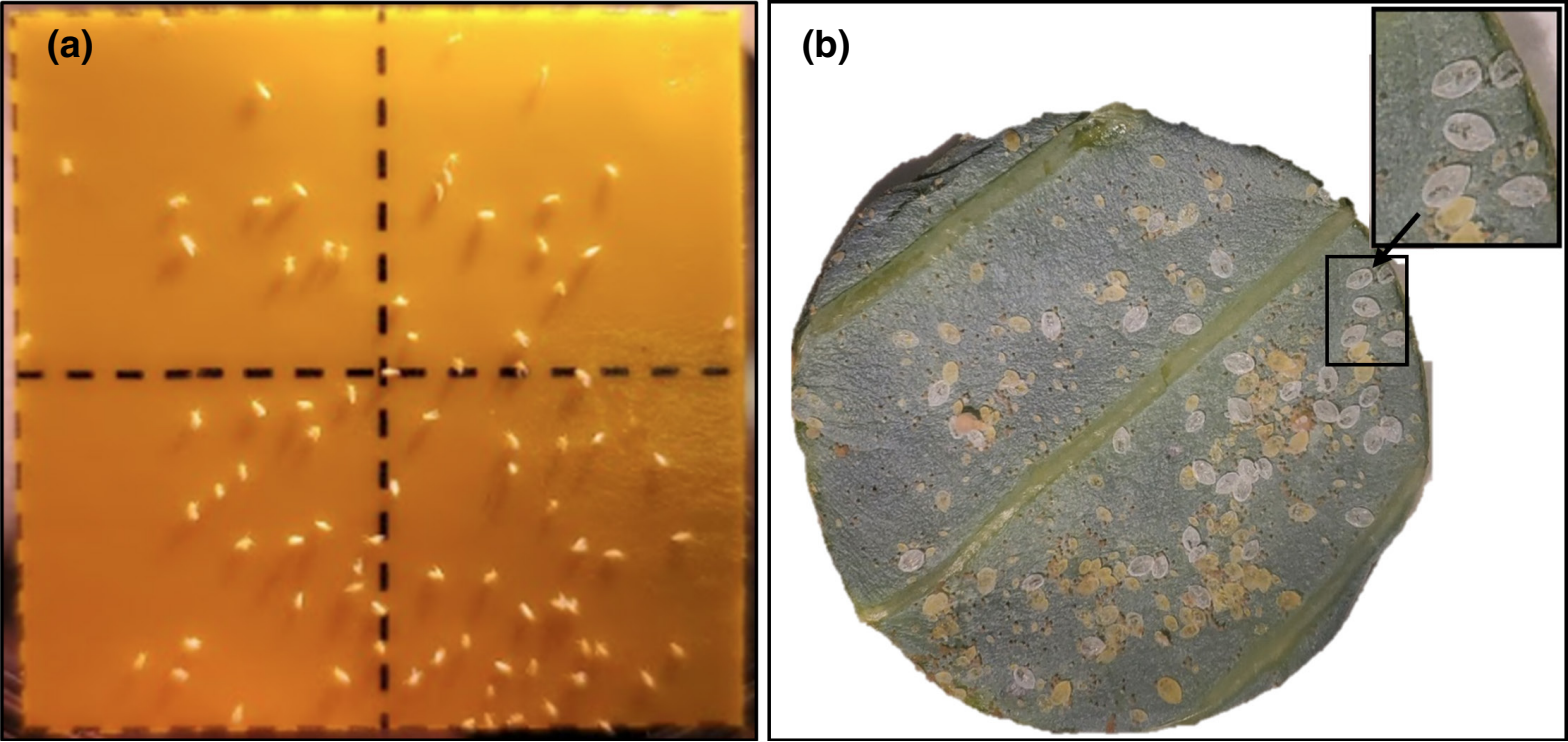
Quality control measures performed for monitoring of whitefly proliferation at 43 days in the colony. (a) Yellow sticky card after 15 min in the cage and (b) leaf punch of a mature ‘median leaf’ from the cage middle plant for counting white nymph exoskeletons per leaf surface area. Current procedure utilizes a wider area leaf image to assure a radius of a circular surface area that has a minimum count of 25 exoskeletons (see Supplemental S5) [Colour figure can be viewed at wileyonlinelibrary.com]

Yellow sticky cards (Luter 20‐pack Dual‐Sided Yellow Sticky, ASIN: B07MWTL63Y) were used outside the cages to trap whitefly adults as both a sentinel and for removal. A quantitative index was created to evaluate whitefly population density in the cages based on the number of whiteflies trapped over a set period on a standardized area of yellow sticky cards. The size, location, duration, and timing of sticky card counting evolved to have a reasonable but not excessive number of whiteflies to count. The refined standard operating procedure (SOP), which is detailed in Supplemental Info S5 involves placing a 4 × 4 cm yellow sticky card onto a 12″ tall table number holder (New Star TBH‐12/23237) as seen in Figure 2. The sticky card is covered with a small plastic bag during placement in the middle of the cage (to prevent premature whitefly capture). After allowing 15 min for the colony cage to settle after this disturbance, the sticky is uncovered for 15 min. The manual whitefly count on the yellow sticky card was conducted with a stereo microscope.

#### 2.5.2 Emerged 4th instar exoskeleton count

Assessments of adult whiteflies based on sticky card count was found to be quite variable. Counting of eggs was undertaken for many months but was found to be tedious and not amenable to image analysis. The various larval instars can be difficult to distinguish; however, once the adults emerge from the 4th instar, they generate a relatively large, distinct high‐contrast white exoskeleton (Figure 2). Initially a circular punch was used to sample a consistent leaf surface area; a refinement to further improve exoskeleton count is to mark the punch area without excision, then recursively image concentric circles of increasing the leaf surface area until a minimum of 25 exoskeletons is measured as a basis of a normalized exoskeleton count per surface area.

#### 2.5.3 Total whitefly count via image analysis

**FIGURE 3:**
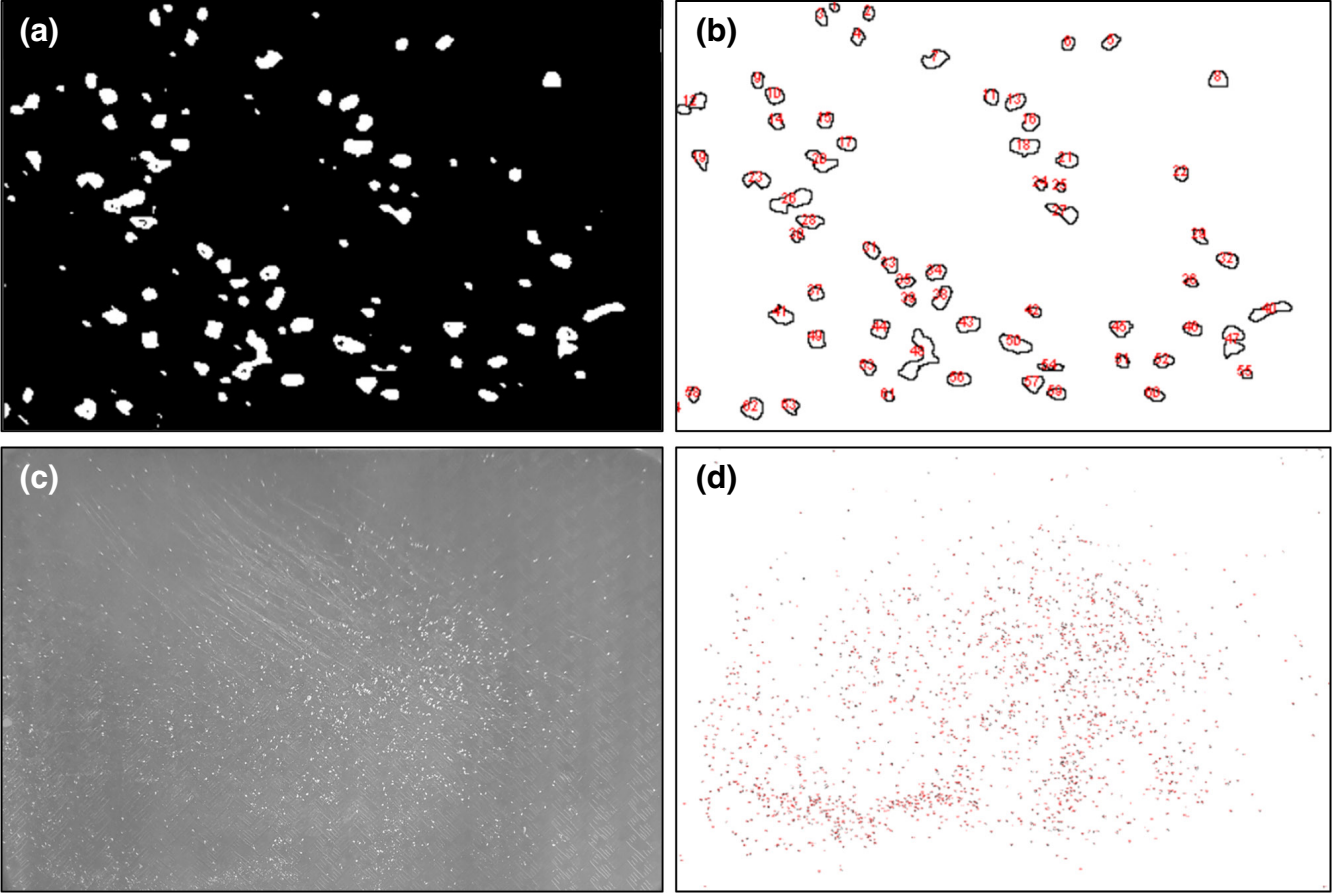
Total harvest whitefly count by image analysis of whiteflies brushed from plants at ~7 weeks (47 days) using ImageJ: (a) small area manual count for validation of automated image count, (b) imaging illustrating automated count of the same 8‐bit image, (c) entire whitefly colony population distributed on a dark surface and (d) automated count of whiteflies in the image using the same colour threshold as the manual validation image [Colour figure can be viewed at wileyonlinelibrary.com]

Counting the whiteflies at 7 weeks (47 days) was performed after the cage was kept at 4°C for at least 48 h to ensure that the whiteflies were immobilized for imaging. The leaves of each plant were excised (for spatial and weight analysis), and the whitefly adults were gently brushed onto a flat dark surface with a very soft 2″ paint brush (Artist'sLoft™ necessities). As shown in Figure 3c, the whiteflies were gently distributed to analyse their number using ImageJ (64‐bit Java 1.8.0_172) (Figure 3d). An independent manual count of a small quadrant of the same image was used to validate and calibrate the automated procedure to within a few percent error (Figure 3a,b). Spraying the whiteflies with water after distribution on the surface was found to dissolve whitefly honeydew and improve image analysis.

### 2.6 Discrete‐time model for whitefly colony proliferation

A discrete‐time model was developed to predict the number of whitefly adults and the health evolution of the plants on a weekly basis in the colony given an initial inoculum amount and plant size. The model follows the predator‐prey model structure. The full list of variables and parameters are presented in Table 1.

**TABLE 1:** Parameters and variables for whitefly colony proliferation model

Variable	Description
P	Number of plants normalized to the initial surface area entering the cage – (P)
A	Total number of viable adult whiteflies – (WF)
T	Total number of adult whiteflies – (viable and dead)
N	Total number of nymphs – (N)
E	Total number of eggs – (E)

Parameter	Value	Description
G_m_	1.34 ± 0.020 P/week	Maximum growth rate of the plants in the cage
K_mx_	0.01	Saturation rate of the plant growth
dt	1 week	The change in time between time points
μ_A_	0.45 ± 0.031 WF dead/WF	Percentage of adult whiteflies that die each week
μ_N_	0.05 ± 0.015 Nymph dead /Nymph	Percentage of nymphs that die each week
μ_E_	0.10 ± 0.008 Egg dead/Egg	Percentage of eggs that die each week
δ	0.45 ± 0.011 Nymph emerge/Nymph	Percentage of nymphs that emerge into adults per week
γ	0.90 ± 0.007 egg hatch/egg	Percentage of eggs that hatch every week
β	0.0000213	Effect of the flies on the plant
r	10.87 ± 2.88 eggs/WF	Number of eggs laid per adult whitefly per week

The health of the plants at any time point is predicted in Equation 1, which quantifies the growth of leaf surface area. The initial surface area, *P_0_*, was quantified on 5‐week‐old cabbage host plants through image analysis.

(1) $$P_{i+1}=P_{i}\;G_{m}‐\beta \;P_{i}\left[A_{i}+\frac{E_{i}}{4}+\frac{N_{i}}{2}\right]$$

where G_m_ is the experimentally determined cabbage plant growth rate under the standardized watering schedule and lighting configuration in the absence of whitefly stress. Parameter β imparts the negative impact of the whiteflies on plant leaf surface area. It is multiplied by the total number of adult whiteflies (*A_i_*), with reduced impacts for eggs ($E_{i}$) and nymphs ($N_{i}$).

The number of living whitefly adults (*A_i_*) of Equation 2 is calculated based on the average lifespan of 16 days for a female whitefly and 10 days for a male (at 28°C). The death rate (*µ_A_*) was initially set based on these literature values to $\mu_{A}=\frac{1}{2}w^{‐1}$ (all die in 2 weeks) and then fit to the experimental data (with whiteflies maintained at 27°C) within the bound region $\mu_{A}\in \left[0.43,0.70\right]$by minimizing the sum square error. Additionally, the rate of development from a nymph to an adult is between 12–16 days at 28°C so was initially set to $\delta =\frac{1}{2}w^{‐1}$(all emerge in 2 weeks) and also fit to the experimental data within the bound region δ∈[0.43,0.58](Aregbesola et al., 2020).

(2) $$A_{i+1}=\frac{\delta \;N_{i}+A_{i}}{1+\mu_{A}}$$

The general colony health, or total whiteflies (*T*), is defined as the sum of living and dead adults as seen in Equation 3. Note that this compartment is a sink. From the definition of $T_{i}=A_{i}+D_{i}$, where *D* denotes the dead files and since $D_{i+1}=D_{i}+\mu_{A}A_{i}$ we can obtain using Equation 2.

(3) $$T_{i+1}=T_{i}+\delta N_{i}$$

The number of nymphs is captured in Equation 4. The development of eggs to nymphs takes about 6–8 days at 28°C so this rate was initially set to $\gamma =1w^{‐1}$ (all hatch in 1 week) and then fit to the experimental data within the bound region γ∈[0.78,1.0] Additionally, as with the adult whiteflies, the nymph death rate ($\mu_{N})$ was initially set based on literature values and then fit to the experimental data within the bound region $\mu_{N}\in \left[0.20,0.40\right]$.

(4) $$N_{i+1}=\frac{\gamma E_{i}+\left(1‐\delta \right)N_{i}}{1+\mu_{N}}$$

The number of eggs at any given week is based on a modified predator‐prey model as presented in Equation 5. At low counts of whiteflies, there is plenty of space to lay eggs and therefore they exhibit unrestrained proliferation. At high counts of whiteflies, space on the plants becomes constrained for both egg laying and feeding, which imposes saturation kinetics on proliferation. The egg‐laying rate (*r*) was determined based on an average total number of eggs laid during a female's lifetime being 109 eggs at 28°C (Aregbesola et al., 2020). Based on the assumption that adults live for 2 weeks, we assume they lay half of their eggs (~50) each week. Finally, this number was divided by two to account for half male whiteflies for the fit to the experimental data. The egg death rate (*µ_E_*) was defined in a similar fashion to the nymphs and adults and finally fit to the experimental data within the bound region $\mu_{E}\in \left[0.20,0.40\right]$.

(5) $$E_{i+1}=\frac{r\left(\frac{P_{i}}{K+P_{i}}\right)A_{i}+\left(1‐\gamma \right)E_{i}}{1+\mu_{E}}$$

Note that although a model that accounts for female and male populations could be created, our experimental observations lack the needed resolution for such a finer description. Additionally, increased numbers of whiteflies deteriorate the health of the plants. Host plant deterioration negatively affects the health of the colony, which is captured in a predator‐prey model by the bilinear term in Equation 5 by the consequence of fewer eggs being laid. While the model captures general characteristics of whitefly and plant growth that could be adapted to more diverse conditions, its utility is intended to provide insight into the system behaviour and specific prediction for colony maintenance.

## 3 RESULTS

### 3.1 Quality control measures

**FIGURE 4:**
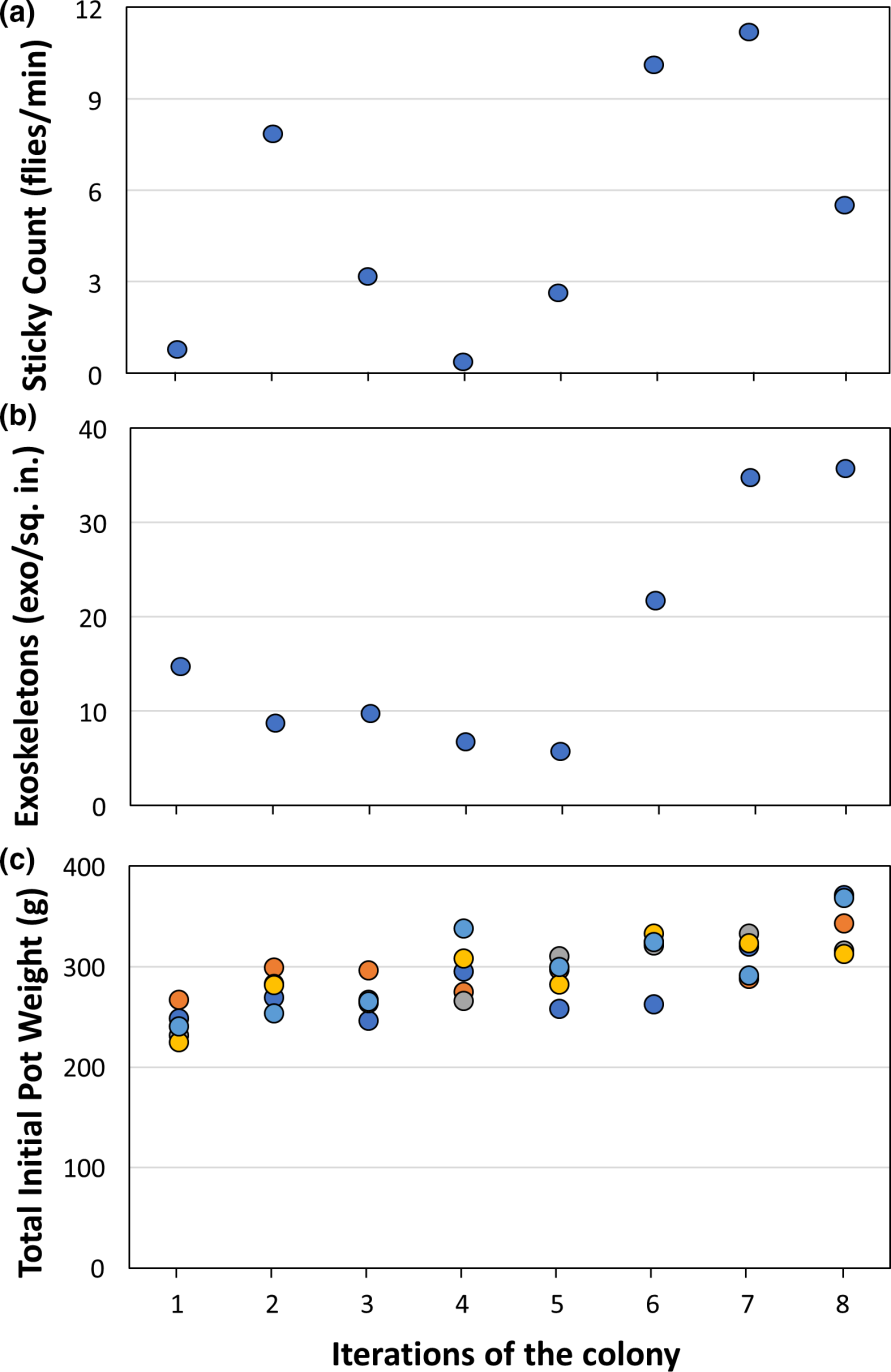
Quality control data taken at the beginning of 7th week over a 2‐month period to quantify variations despite procedural consistency: (a) whiteflies per sq. in. per min stuck on yellow sticky card normalized to the capture duration interval, (b) number of exoskeletons per sq. in. on the ‘median leaf’ using concentric circles around a normalized punch and (c) initial potted plant weights entering the colony [Colour figure can be viewed at wileyonlinelibrary.com]

The goal of achieving a compact whitefly colony that occupies roughly 20 square feet of laboratory space and relatively inexpensive to maintain was accomplished and could consistently provide hundreds to thousands of whiteflies for experimentation on a bi‐weekly basis. However, despite the focus on consistency of inputs, and a qualitatively healthy colony productivity, the quantitative outputs reflected considerable instability. Numbers of exoskeletons and adult whiteflies at the 7th week of colony proliferation reflected a consistent correlation in the variation of these productivity indices. The number of whiteflies caught on the 16 cm^2^ yellow sticky cards in 15 min ranged from 1 to 11 (Figure 4a) while the exoskeletons varied from 6 to 37 (Figure 4b) per 0.78 in^2^ (Thompson et al., 2022 ). Although these variations are much smaller than initial efforts that included complete death of some plants, they illustrate the challenge of achieving consistency in a scaled‐down colony despite extensive systematic methodologies.

**FIGURE 5:**
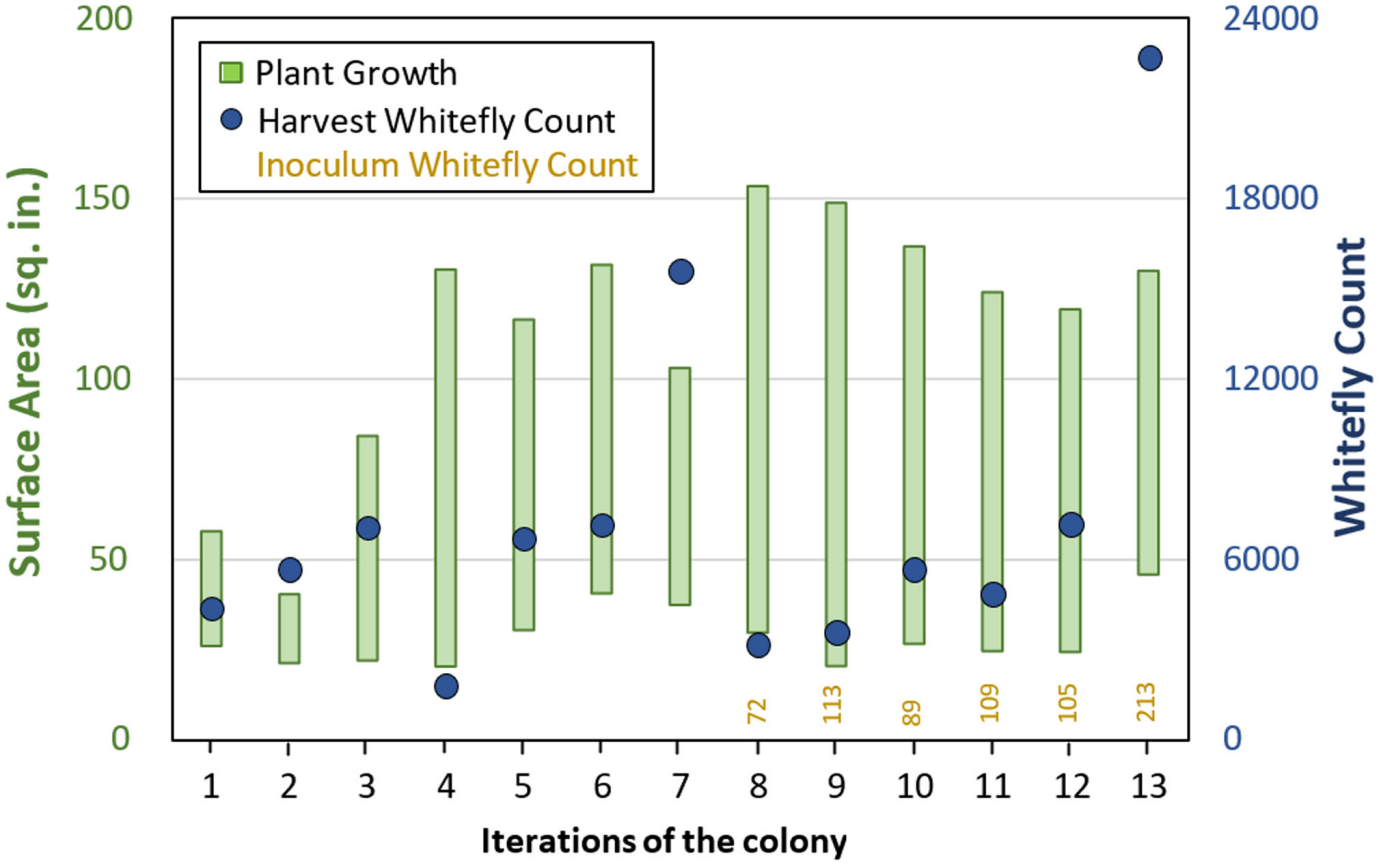
Iterations of colony performance. Average plant growth (total leaf surface area per plant) and corresponding end‐point whitefly count at successive iterations of colony maintenance. The green bars represent growth as plant surface area between inoculation week 0 in the colony (bottom) and harvest at 47 days (top). Blue circles indicate the total whitefly count at 47‐day harvest. Quantitative whitefly inoculation numbers (using single leaf inoculation method) are provided above the iteration number. Final data point (iteration 13) indicates the high inoculum test of an initial 213 whiteflies, which resulted in a harvest of 22,740 whiteflies [Colour figure can be viewed at wileyonlinelibrary.com]

Total initial pot weights (Figure 4c) and the initial leaf surface area (Figure 5) of inoculated host plants showed a slight increase as our methods of growing cabbage improved where leaf surface area was observed to be a reasonably consistent, quantitative indicator of initial host plant size. The plant leaf surface area at the time of harvest showed that plant size had increased by roughly 4‐fold during whitefly proliferation (Figure 5), while there did not appear to be a correlation between initial and final plant surface area – likely limited by lighted growth area within the cages. This is corroborated by an additional three colony iterations conducted with four cabbage plants as compared to five. No significant difference was observed in either average final total leaf surface area or the total whiteflies produced (*p* = 0.43 and *p* = 0.47 respectively) with an additional reduction in materials and ease of maintenance for four cabbage plants. Finally, the overall total whitefly harvest experienced counts up to 15,000 whiteflies (Figure 5). As expected, there was a negative correlation between number of whiteflies and final plant surface area that is less evident at lower populations of whiteflies (e.g. below 10 k). An inoculation with roughly double the initial whiteflies (~200) illustrates the detrimental impact on the plants (Figure 5, Iteration 13; Supplemental Figure S8‐3) that results from the high amplification ratio of whiteflies and systemic stress gene expression that results from whitefly feeding (Ogden et al., 2020).

### 3.2 Model results

**FIGURE 6:**
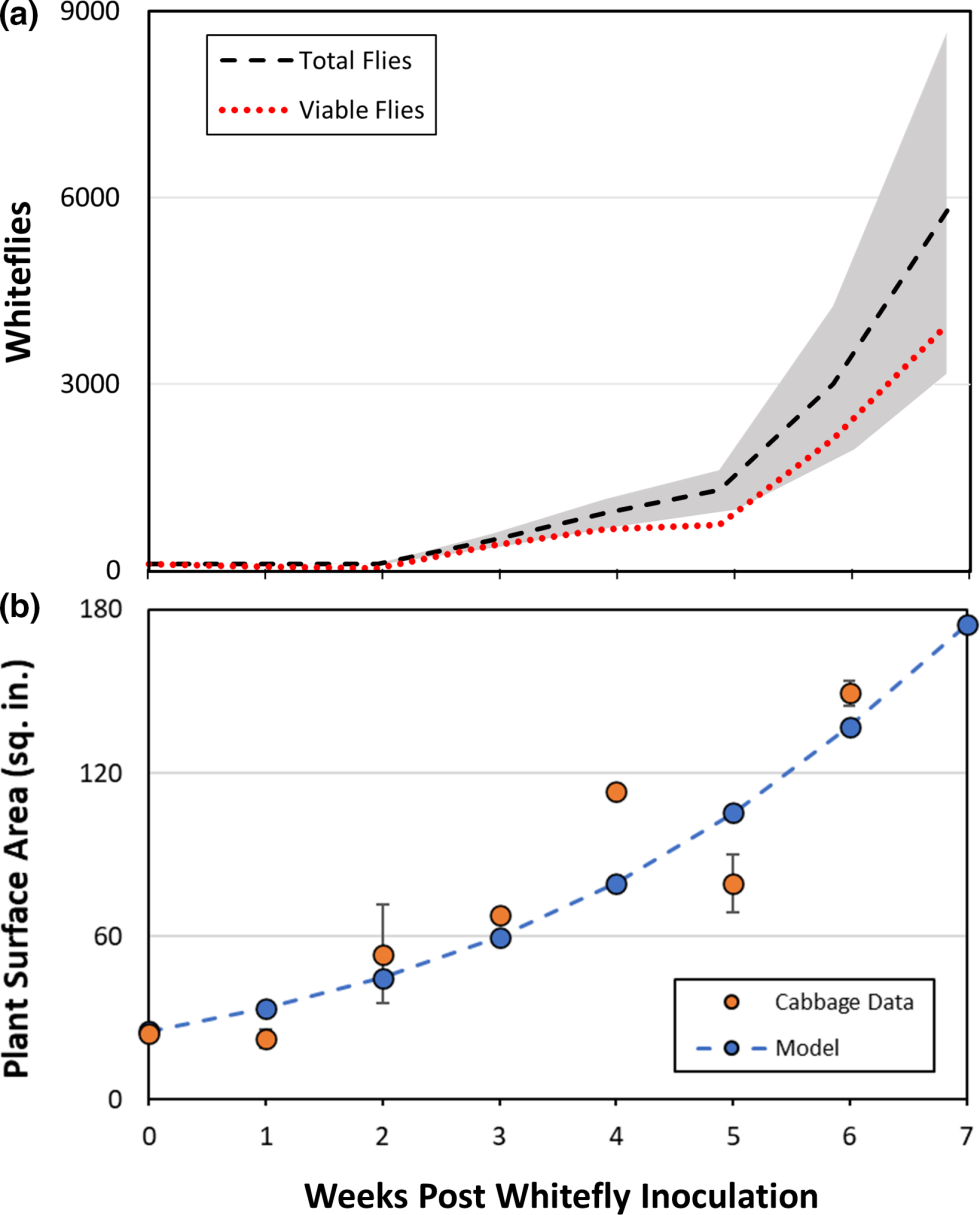
Model predictions of whitefly colony behaviour over 6 weeks after inoculation showing (a) modelling of total adult whiteflies with an initial inoculum of 100 whiteflies with grey area indicating the standard deviation (Table 1). Viable (alive) adult whiteflies are indicated as the red dotted line. Harvest occurs at 47 days and does not distinguish dead vs alive whitefly count; (b) experimental and modelled growth of a single average cabbage plant after entering the colony. Plant growth is measured as surface area of the leaves experimentally computed from image analysis (see Supplemental Info S7) [Colour figure can be viewed at wileyonlinelibrary.com]

The model parameters fit to the experimental data of total harvested whiteflies (Figure 5, iterations #8–13) and plant surface area are presented in Table 1 and calculated predictions presented for an inoculation of 100 whiteflies in Figure 6. The delay period in total adult whiteflies from week zero to two reflects the hatching of eggs to nymphs. The subsequent increase in the viable adult count was dramatic due to the 1:80 amplification ratio for the adult females. This high amplification ratio also results in the vast majority of whiteflies being viable as indicated by the dotted red line of Figure 6a being comparable to the total. Viable whiteflies decrease from weeks 0–2 due to adult whiteflies dying while the eggs/nymphs develop. The growth of cabbage plants without whiteflies was a simple fit to experimental data; plant surface area increased 4‐fold over the 6‐week period required for two whitefly life cycles. A one‐way analysis of variance was conducted on the plant growth model to demonstrate legitimacy of the model by comparing the model predictions with experimental results. It assessed the model as significant (*F*_calc_ = 97.5 ≫ *F*_table_ = 18.5), and an *F*‐Test on the Left‐Out‐Terms (LOT = residual − replicate error; Trauger et al., 2021) resulted in *F*_calc_ = 1.62 < *F*_table,0.05_ = 5.59; thus, we conclude an adequate fit of model at greater than 95% confidence.

**FIGURE 7:**
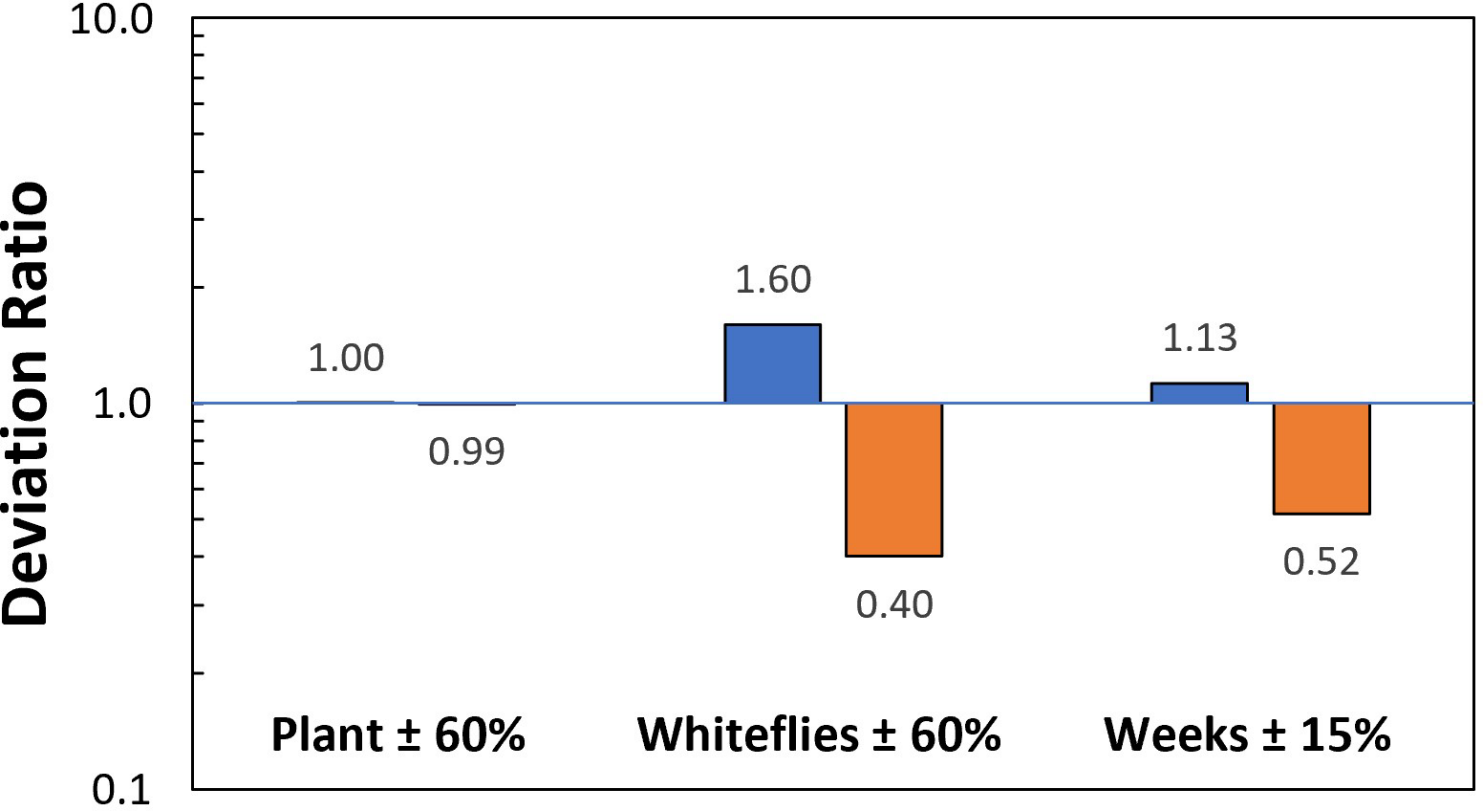
Whitefly production model deviation analysis for the predicted total whitefly population relative to the standard condition: initial leaf area of 25 sq.in. per plant, 100 whitefly inoculum and 7 week harvest duration. The assessment is the deviation ratio in predicted total harvested whitefly in response to 60% changes in the plant leaf surface area, whitefly inoculation and 15% duration (±1 week) in the harvest duration. Consistent with qualitative observations over several years, initial whiteflies and the duration of colony amplification have a far greater impact on the final whitefly count than the plant size [Colour figure can be viewed at wileyonlinelibrary.com]

To assess the sensitivity of the colony performance, a deviation analysis was conducted on the three main variables (input leaf surface area, input whitefly count and proliferation time) and is presented in Figure 7. The change in the final total whitefly count due to an increase of 60% in plant leaf surface area (i.e. plant size) is minimal compared to whitefly inoculation count or proliferation duration (no change = 1). The 60% deviation percentage in initial whitefly count is within the range of possibility for a bias of predominantly male or female inoculum whiteflies. The strong impact of proliferation time that results from logarithmic whitefly proliferation is apparent, as a comparable deviation in total whitefly output to variations in whitefly number inoculation is achieved for only an increase or decrease of one week (15% deviation) in proliferation time.

## 4 DISCUSSION

Preliminary efforts at establishing a whitefly colony at Penn State in a walk‐in growth chamber with plants initiated at a greenhouse had extensive problems including humidity control and the associated mildew contamination, resulting in poor colony health. In trying to replicate a comparable whitefly colony capability at the new performance site, the original project costs for technician time, heavily subsidized greenhouse and walk‐in incubator space were in excess of $100K U.S. per year. This led to a complete ‘re‐engineering’ of the approach to colony maintenance to improve whitefly productivity while reducing cost with the goal of a whitefly colony that could be maintained within a typical laboratory space that did not have dedicated plant growth infrastructure or technical maintenance personnel. Reducing whitefly colony initiation to a bi‐weekly basis (after more than a year of weekly maintenance) was a simple substantial reduction in space and materials that had minimal impact on whitefly availability for experimentation and balanced efforts for predominantly plant and predominantly insect manipulation as a convenience for colony workflow. The scaled‐down implementation described here represents a high‐performance whitefly colony (~5000 whiteflies bi‐weekly) that costs an order of magnitude less, with capital costs (incubator cages, plant growth materials) of about $25K U.S. and minimal materials and operation costs, which do not require the extensive infrastructure and overhead of growth rooms and greenhouse facilities. Near complete biocontainment of the whiteflies was also observed based on observations during manipulations, sticky cards both inside and outside the incubator, and unprotected ‘indicator plants’ outside the incubator.

A discrete‐time interval‐based model combined with parameterization constraints from the literature of whitefly reproduction provides a descriptive model that is sufficiently simple for others to use for similar scaling whitefly production to experimental needs. Despite the highly controlled environmental conditions and quality control measures, the exponential nature of this growth model and its ±25% standard deviation in accumulated whiteflies for two whitefly life cycles are illustrative of the challenge of modelling whitefly proliferation. Besides the protracted effort and difficulty of obtaining data for model refinement, the predator‐prey nature of the whitefly‐host plant interaction is fundamentally unstable, with the instability becoming greater for smaller populations (Tahara et al., 2018). It should be kept in mind, that classic biological models of growth for suspensions of cells, a 1 ml inoculum of optical density of 1 will contain on the order of 10‐million cells, which typically grow by simple division and allow for nearly continuous time point measurements (Myers et al., 2013). Refinements in fecundity and death rates are far more difficult to assess; none‐the‐less, obtaining more refined models and experimental platforms could be invaluable for assessing very different strategies of insect control such as reducing fecundity, attenuating lifespan, male sterility etc.

**FIGURE 8:**
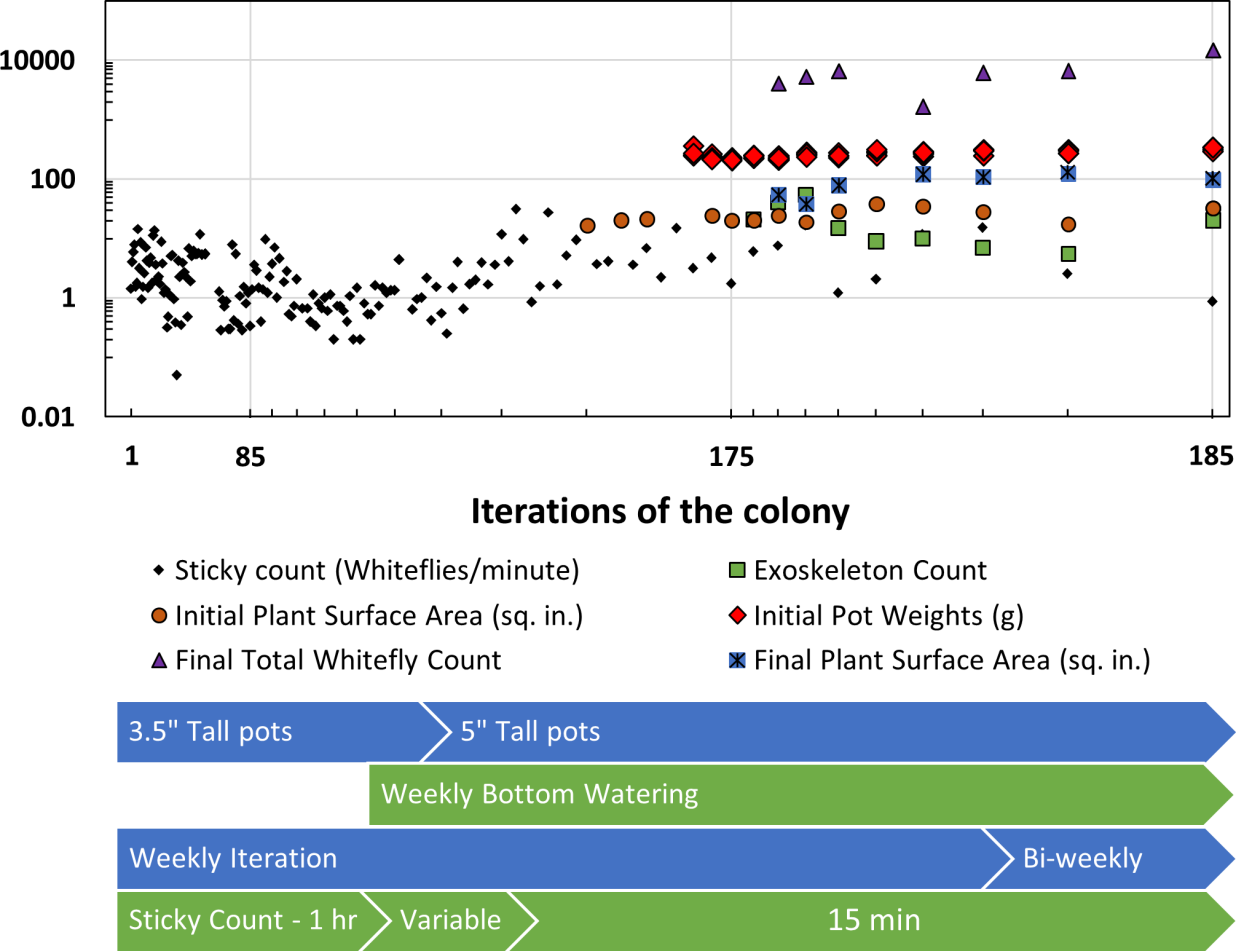
Timeline of quality control measures and colony implementation changes. The sticky count indicator of whitefly capture rate was the initial focus for feedback changes to colony implementation. During that period (I < 170) large variations in plant health and whitefly count was observed – including near complete colony collapse. Additional quality control measures were incorporated as the process was improved and refined (I > 170). The graph illustrates the difficulty of achieving stability in this small‐scale system and ultimately the effectiveness of quantitative inoculation [Colour figure can be viewed at wileyonlinelibrary.com]

Since the goal in this work was to dramatically scale down the colony (in both size and effort), the SOP was iteratively refined as we sought improvements. Feedback for changes were informed by qualitative observations and quantitative quality control measurements. Monitoring the health of the colony through sticky‐paper counts, exoskeleton counts and total whitefly counts allowed us to troubleshoot and implement a qualitative feedback control methodology. Some discussion on this evolution of methods is informative towards future improvements. A problem encountered in scaling down to small lab‐grown host plants was frequent plant collapse due to too many whiteflies or inadequate proliferation due to too few inoculum whiteflies or unhealthy plants. In the initial scaled‐down reach‐in incubator configuration, one of the five cabbage host plants entering the colony was chosen to be the inoculum plant and placed inside of the oldest cage for 48 h over the weekend. After this whitefly exposure period, the transfer plant was captured within a paper bag and transferred to the new cage, which was subsequently refined to an acrylic transfer tube (see Supplemental Info S4). This protocol was in use for the first 160 weeks and corresponded to a highly variable whitefly sticky count (Figure 8). An unstable cycling was observed, where the colony would build to better whitefly numbers, and then experience colony collapse as anticipated for an unstable predator‐prey relationship. To overcome over‐inoculation, the inoculum acquisition time was scaled back to 1 h and then 15 min but we still did not observe a stable performance as the number of whiteflies entering the colony would vary depending on the health of the iteration it was inoculated from. For example, from Figure 4, the total count of week 4 was shown to be dramatically lower than previous weeks. Looking through the QC logs, this correlated with a particularly low inoculation number (back‐calculated from the model corresponds to between 20 and 30 whiteflies). With this observation of a strong sensitivity to whitefly inoculum counts, a quantitative inoculation based on known whitefly numbers was developed to avoid acquisition based on time. This was accomplished while avoiding highly accurate but tedious methods for whitefly manipulation, for example aspirator counting (Polston & Capobianco, 2013).

This experience with colony maintenance and model predictions converged to a target inoculum count of between 80 and 110 whiteflies. An inherent assumption of our inoculation method is that the procedure acquires random males and females; however, this may be biased towards a greater number of males due to being more mobile relative to the larger feeding and egg‐laying females. It is noteworthy that for extended acquisition times, when fewer whiteflies were available in the inoculum cage, there was a clear pairing of males and females (see Supplemental Figure S8‐4) that is not apparent when higher whitefly loads in the inoculum source cage resulted in more rapid acquisition. Such a female/male biasing provided by this inoculation method could be expected to help stabilize colony performance. While a refinement based on sexing of whiteflies based on size from image analysis might provide more accurate modelling, we wanted to retain the simplicity of total whitefly inoculum of ~100. More whiteflies than this overloads the host plants and rapidly deteriorates their health. To confirm this, an inoculation with 200 whiteflies was undertaken. As seen in Figure 5, iteration 13 corresponds to this high inoculum and it resulted in a final count of 22,740 whiteflies and dramatically lowered plant health that was clearly evident by 4 weeks for this proliferation cycle (see Supplemental Figure S8‐3).

While the focus of whitefly colony maintenance is to provide adult whiteflies, maintaining the health of the host plants was observed to have a large impact on whitefly proliferation as predicted by any predator‐prey model. Numerous improvements were adopted to improve plant health such that eventually, plant growth was largely constrained by the cage ‘footprint’. The soil volume was increased by increasing the pot height from 3.5″ to 5″ to improve (a) space for root system growth and (b) consistency of water availability. Related to water availability, it was observed that weekly sub‐irrigation with at least 30 ml of water per plant prevented older plant wilting and sustained cabbage plant health. Notably, this was coordinated with weekly (Wednesday) sub‐irrigation with Gnatrol in the cabbage growth ‘pipeline’ outside of the colony during the 5‐week growth period of the host plants. The use of the automated watering peristaltic pump with a remote‐control switch was an invaluable asset to avoid over‐watering and make this daily task minimal – furthering the goal of streamlining and cost (time) reduction. Consistent with observation, the model is rather insensitive to plant growth – in part because of visual adjustments to avoid obviously detrimental plant health. Noting that the surface area of the cage is 288 in^2^ as compared to an average final leaf area of 625 in^2^, more than half of the leaf surface area is not directly exposed to the PAR light levels of ~110 µE m^−2^ s^−1^ measured inside the cage at mid‐height between pot and cage top. Given the improvements in plant growth and the associated observation of light limitation, the use of only four cabbage plants is sufficient for the inoculum of roughly 100 whiteflies – thereby further reducing time and materials for maintenance while still resulting in a production of around 5000 whiteflies.

Overcoming problems of scaled‐down plant and whitefly performance was approached by moving from qualitative to quantitative monitoring a variety of inputs ranging from pre‐colony plant growth to soil volume and moisture as they related to total whitefly performance indices after nearly 7 weeks of proliferation. We were able to create a cost‐effective high‐performance approach to a whitefly colony maintenance that can reduce the burden and investment required for researchers to execute whitefly research and thus address a critically important aspect of crop protection and viral disease transmission research.

## Conflict of Interest Statement

The authors have no conflicts of interest.

## AUTHOR CONTRIBUTIONS

Natalie Thompson and Wayne Curtis conceived the research. Natalie Thompson, Nadia Waterton and Wayne Curtis conducted experiments. Jane E. Polston contributed material, experience and advice. Natalie Thompson, Antonios Armaou and Wayne Curtis analysed and modelled data and conducted statistical analyses. Natalie Thompson and Wayne Curtis drafted the manuscript. Antonios Armaou, Jane E. Polston and Wayne Curtis secured funding. All authors read, edited and approved the manuscript.

## Supporting information

Supplementary Material

## Data Availability

The data that support the findings of this study are openly available in Penn State Data Commons (https://doi.org/10.26208/kfkk‐yz58).
