# Supplementary material for: Establishing an inexpensive, space efficient colony of Bemisia tabaci MEAM1 utilizing modelling and feedback control principles
Source: J Appl Entomol. 2022 Mar 24;146(5):648–58. doi: 10.1111/jen.12995 (PMC9544070; doi:10.1111/jen.12995)
Supplement: Supplementary file 1 — Supplementary Material [file JEN-146-648-s001.docx]

**Supplemental Information**

Establishing an inexpensive, space efficient colony of *Bemisia tabaci* MEAM1 utilizing modeling and feedback control principles

Authors:

Natalie Thompson^1^, Nadia Waterton^1^, Antonios Armaou^1,3^, Jane Polston^2^, Wayne Curtis^1^

(1) Department of Chemical Engineering, The Pennsylvania State University

(2) Department of Plant Pathology, University of Florida

(3) Department of Mechanical Engineering, The Pennsylvania State University

**Table of Contents**

[S1 – Watering Cart Description 2](#_Toc80798238)

[S2 – Host Plant Growth and Colony Infrastructure 5](#_Toc80798239)

[S2.1 Cabbage host plant growth area 5](#_Toc80798240)

[S2.2 Whitefly colony incubator 5](#_Toc80798241)

[S3 - Whitefly Quantitative Inoculation Protocol 8](#_Toc80798242)

[S4 - Whitefly Qualitative Inoculation Protocol 10](#_Toc80798243)

[S5 – Quality Control Procedures 12](#_Toc80798244)

[S6 – Scaling Transfer from Small Colony 15](#_Toc80798245)

[S7 – Image Analysis 16](#_Toc80798246)

[1 – Leaf Surface Area 16](#_Toc80798247)

[2 – Whitefly Cage Harvest Count 16](#_Toc80798248)

[S8 – Images 18](#_Toc80798249)

# S1 – Watering Cart Description

Overview**:** The addition of a watering cart facilitated several important implementation components for whitefly colony maintenance. The use of a peristaltic pump facilitated reproducible delivery of water and fertilizer solutions. Using a remote control on/off switch allowed for very rapid and precise water delivery to either the surface or the bottom of plants. The upper vinyl cage panel allowed visual observation while moving watering between plants, and water delivery at the end of a road prevented the escape of whiteflies (as the watering tube passed readily through the ‘sock’ in the insect cage door).

**Construction:** While the implementation of a watering cart can have various permutations, we provide our fabricated solution here due to its usefulness in size, maneuverability, storage of water, and placement of the rod / tubing extension for simple ethanol spray cleaning between cages. The watering cart is shown schematically in Figure S1-2, and a picture in Figure S1-3 with an inset close-up of fabrication parts. Our implementation was constructed from 45x45 mm T-slot extruded aluminum framework with 1.5” corner gussets (T-nutz.com; Part # CB-040-A) which included bracing for shelves to support 1/8” plastic shelves (US Plastics, Item # 45282). The top shelf is 32”x12”x½” hardwood butcherblock laminate. For maneuverability, the front wheels are fixed position (T-nutz.com; Part # CAS-015-R) and the rear are swivel (T-nutz.com; Part # CAS-015-S). A positive displacement peristaltic pump was utilized to provide precise flows (e.g., Watson-Marlow 5025) on the lowest shelf. It uses a 0.5” outer diameter (OD) tubing (e.g., PharMed Tubing; Item # 57321) to pump water from a gallon jug that rests 11” above the pump to an adaptor which adjusts the tube size to a 0.25” OD silicone tube (e.g., MasterFlex 6492-18 0.170” ID x ¼” OD). Note that the 11” of pressure head above the pump is beneficial to reduce bubble entrainment and siphon issues. The watering tube is attached to a 36” long 0.25” DIA aluminum rod which provides rigidity when entering the tube through the fabric sock into a cage. On/off control of the peristaltic pump is easily managed with a remote-control plug-in outlet (Intertek, #UTTNOREM2), allowing for precise watering of individual plants.

Calibration of the volume of water exiting the watering tube based on the number of seconds that the pump was on was performed to ensure consistency. These results can be seen in Figure S1-1 to be highly consistent at 5.86 ± 0.04 mL/s. For conditions of our lab, daily watering started at 8s (weeks 1 to 2) and increased to 12s by weeks 5 to 6.


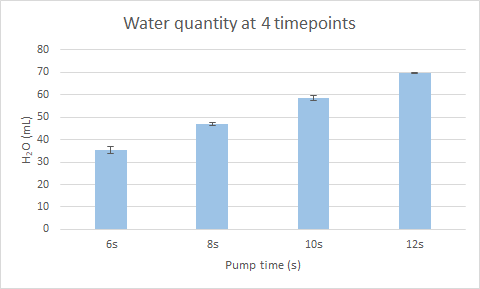


**Figure S1-1**: Consistency of water pump delivery. Volume of water leaving the pump after 6, 8, 10, and 12 seconds was measured by mass with data points taken in quintuplet.


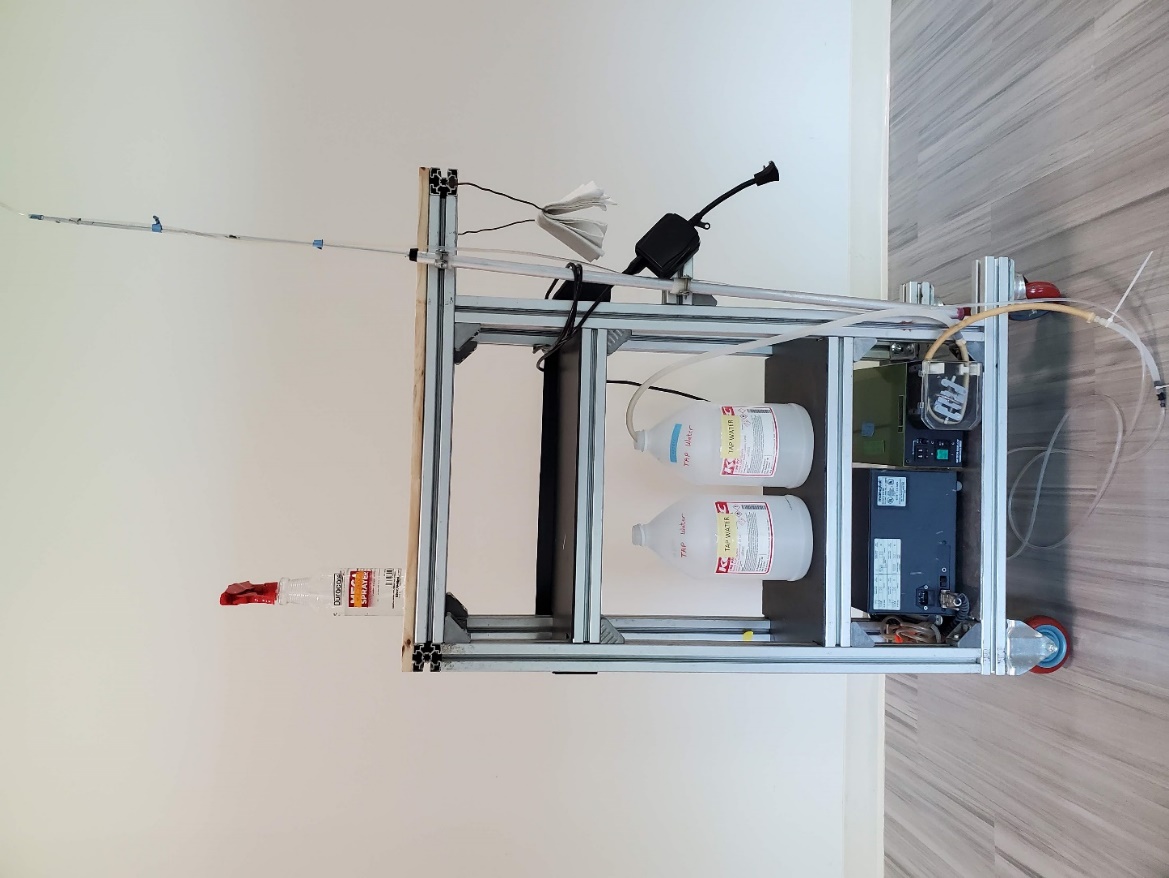

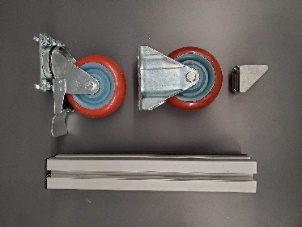


D

A

B

C

A

B

C

D

**Figure S1-3**: Picture of the watering cart. Close-up of the individual parts is seen in the top left with (A) the 45x45mm T-frame, (B) the back movable wheels, (C) the front fixed wheels, and (D) the 1.5" corner gussets

H_2_O

45”


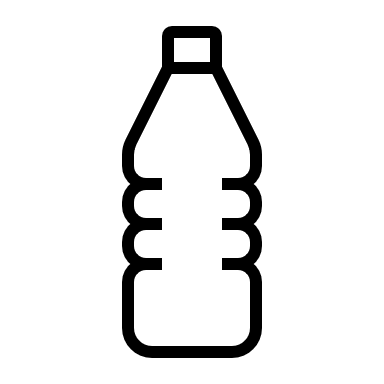


**Figure S1-2**: Schematic of the watering cart design consisting of a peristaltic pump below a gallon jug of water which pumps water up through an extension that is used to direct flow to individual plants.

7”

18”

35”

32”

2’

# S2 – Host Plant Growth and Colony Infrastructure

The growth of the plants in a non-greenhouse, laboratory setting provides for more reproducible growth conditions but necessitates the use of artificial lighting. For whitefly colony maintenance, there are two growth areas: (1) the ‘whitefly free’ location of the initial cabbage host plant growth area prior to being introduced into the colony and (2) the whitefly colony incubator itself.

## S2.1 Cabbage host plant growth area

The cabbages are planted and maintained in a 24x24x24” BioQUIP screen cage as seen in Figure S2-2. In most recent implementation, the cage lit by 6 fluorescent tube lights (Ecolux, SPX41-F32T8) in a 6-Light High Bay Ballast (Item #: 1034047, Model #: IBZT8 6), placed 3” above the cage. The lights are set to a schedule of 16 hours on – 8 hours off using automated TORX timers. The PAR light levels measured at the 5” pot height in this configuration are roughly 110 μmoles m^-2^ s^-1^. A schematic of the set-up is shown in Figure S2-1. The most recent implimentation is on a custom built free-standing rack with a 28x57x½” plywood shelf that is supported by Unistrut channel (Part #: A1001, 14 Gage Channel, Back-to-Back) and Unistrut bracket (Part #: A2494, 12” Bracket). Note that the use of cabbage as a non-viral host for our work could accommodate much shorter growth cage; however, our configuration was found to be very versitile for the growth of other plants including dwarf Tomato (Fla. Lanai) and *Nicotiana benthamiana*.

A simpler bench-top implimentation of lighting that was initially used is also briefly described. This involved hanging a quad spot-light above the cage fabricated from materials readily available at local home supplies as pictured in Figure S2-3. A parts list and step-by-step tutorial for assembly is available on the lab web page or upon request. Key parts include:

1. Square conduit ceiling box
2. (4) offset nipple wiring conduits (Halex 90401).
3. (4) red-dot s500e lampholder
4. (4) 19W LED 25^o^ spot lights (CREE TPAR38 3000K)

## S2.2 Whitefly colony incubator

The whitefly colony incubator (SP Scientific, Hotpack 33 cubic foot chamber, Part #: 100000583) facilitates four cages split between two levels as seen in Figure S2-4. Each level has three LED fixtures (Beams Work DA FSPEC LED Aquarium - 30”, Model # DA80) that are spaced evenly across a total depth of 26” and held 23” above the shelf or 5” above the top of the 18” tall whitefly cage. This configuration provides a PAR light level measured inside the cage at the 5” pot height of roughly 110 μmoles m^-2^ s^-1^. The photoperiod is set to a schedule of 16 hours on – 8 hours off using timer modules (Odyssea, Part #: 9274238, ASIN: B00EDMOHH8). Note that we found the OEM LED power supplies for these LED fixtures to fail relatively quickly relative to the LED life, and found an alternative power supply to be available for their systematic failure (PK-power, 15V 3A male #B06XQKKTZV).

48”


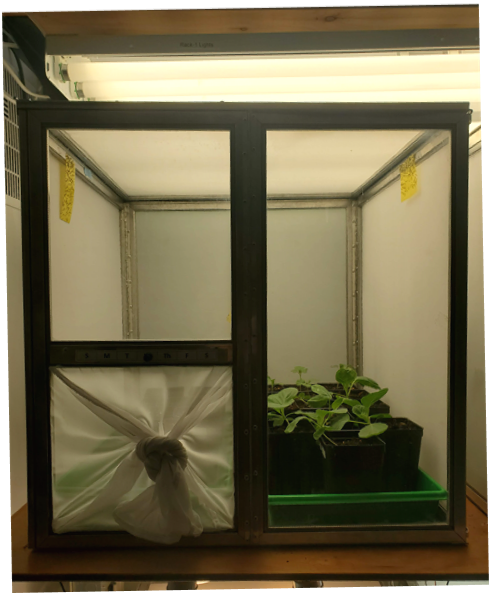


**Figure S2-1:** Schematic of the whitefly-free growth cage for the cabbage host plants on a shelf with 48” lights. The size of the shelf and lights facilitates an extra 24” cube cage for spare plants if needed.

**Figure S2‑2:** Picture of the BioQUIP cage for the growth of the cabbage host plants (ambient lab conditions).

28”

57”

27”

24”

24”

24”


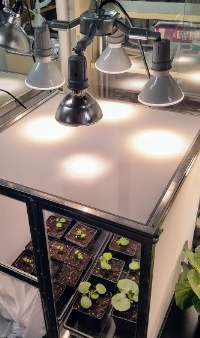


Figure S1-C: Watering Cart consistency measurements. Volume of water leaving the pump after 6, 8, 10, and 12 seconds was measured by mass with data points taken in quintuplet.

**Figure S2-3:** Picture of alternative benchtop host plant production cage with LED spotlight configuration

**Figure S2-4:** The whitefly colony growth chamber containing four screen cages. (a) Schematic of the set-up showing dimensions and (b) a picture of the interior of the whitefly colony incubator.

23”

12 ¼”

24”

18”

30”

23”


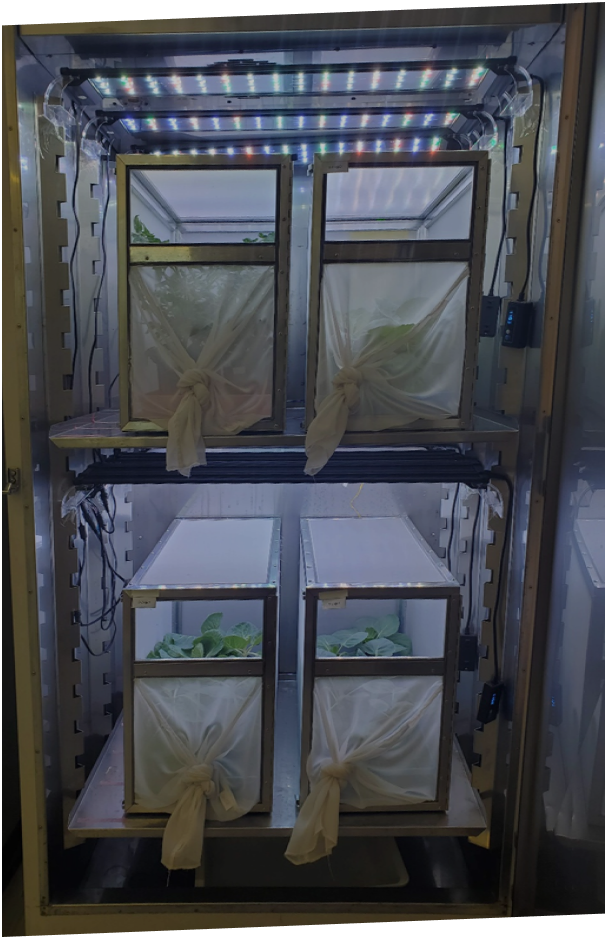


**b)**

**a)**

# S3 - Whitefly **Quantitative** Inoculation Protocol

Basic procedure to inoculate a new cage with a **known quantity** of flies. The ability to quantitatively inoculate each new colony ensures consistent results in the health of the colony and availability of reasonably synchronized whiteflies.

1. **Prepare host plants**:
   1. Set up five 5-week-old cabbage seedling plants every other Monday in a cleaned and dry cage. Note: a pink 1020 greenhouse tray bottom provides valuable contrast for image analysis.
   2. Take a picture from directly above of the plants arranged on the flat with a ruler for initial surface area measurement as seen in Figure S3-1a.
   3. Measure the weight of each plant **before** watering: If total plant weight is less than 300 g, add up to 30 mL of water.
   4. Place the plant pots on a ‘yogurt lid’ to provide for bottom watering spaced out on a 1020 greenhouse flat tray, into the cage in the whitefly growth chamber incubator.
2. **Prepare inoculum Monday afternoon of same week**:
   1. Select a healthy 5-week-old cabbage plant.
      1. Cut off all but one of its leaves (which must be small enough to fit into a 90 mm petri dish) and prop it up with a stick (e.g. Puritan Medical Products, 6in. #807) to be able to see the underside as seen in Figure S3-1b
   2. Place inoculum plant in a mature colony cage to obtain the whiteflies.
      1. The cage that has completed 6^th^ / beginning of 7^th^ week will be in its second generation of whitefly emergence.
      2. Place the plant on top of an elevated surface (such as an empty inverted pot); the goal is to have this inoculum leaf above other plant leaves to help attract whiteflies.
   3. Capture ~100 whiteflies on the inoculum leaf.
      1. The number of flies can be assessed by taking pictures; A high contrast picture can be obtained using oblique lighting with a spotlight (and temporarily turning off overhead light for the picture). The number of flies is then easily counted manually from the picture as seen in Figure S3-1c.
      2. The accumulation of whiteflies can take anywhere from several minutes to several hours: check often and keep track of time. The majority of whiteflies will move to the bottom side of the cabbage leaf if given sufficient time. Performing this task mid-day (during more active whitefly movement) seems to be helpful. Another helpful action is to place a yellow piece of paper on the outside of the cage close to the inoculum leaf to attract the whiteflies and then subsequently remove the paper to encourage them to move to the inoculum plant leaf.
      3. Excise the leaf into a 90 mm petri-dish; this can be done quickly & carefully to avoid whitefly escape, by using both arms through the cage sock to hold the top and bottom of the petri dish and sandwiching the leaf into the petri dish using the edges to cut off the leaf. Note that wiping the petri dish with an anti-static dryer sheet largely eliminates the problem of whiteflies being trapped to the plastic by static charge.
3. **Introduce the petri-dish containing the leaf and flies into the new cage; remove the cap & release the flies**.
4. **Iterate the cage numbers**.
   1. The use of movable stickers to indicate the length of time that the cage has spent in the colony eliminates confusion and ensures a smooth iterative process. Our nomenclature is as follows:
      1. At the time of whitefly inoculation, the new cage becomes **Cage 1-2** (having spent 0-2 weeks in the colony), and each subsequent cage is likewise iterated. For example, **Cage 3-4** (having spent 3-4 weeks in the colony) is starting its 5^th^ week and therefore becomes **Cage 5-6.**  Finally, the old **Cage 5-6** is starting its 7^th^ week and therefore becomes **Cage 7-8** which will undergo QC (quality control) analysis the next day on Tuesday.

**Figure S3-1:** Whitefly inoculation procedure (a) where the five plants entering the new cage (Cage 1-2) are photographed from above for surface area measurements, (b) one plant is chosen to be the inoculation plant has all but one of its leaves removed, and (c) the number of flies on the inoculation leaf is quantitatively observed through lit photos of the underside of the leaf.


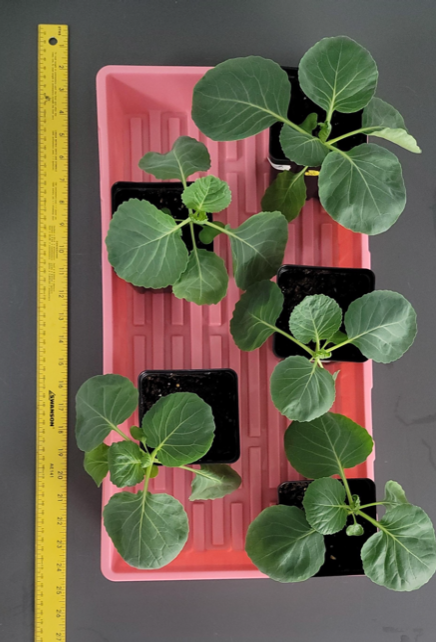

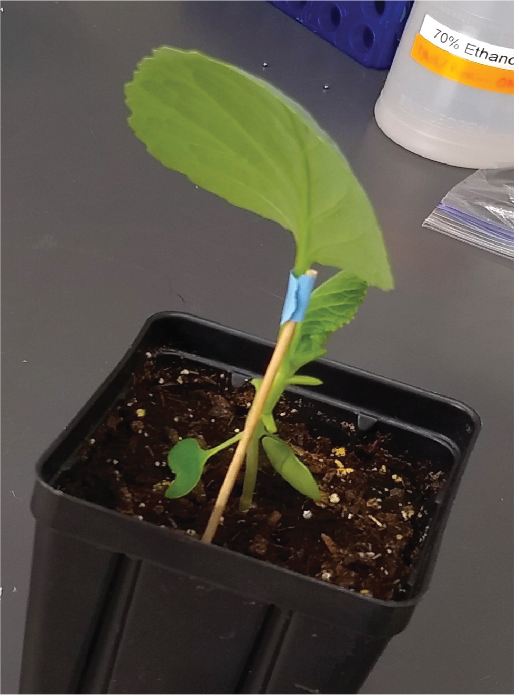

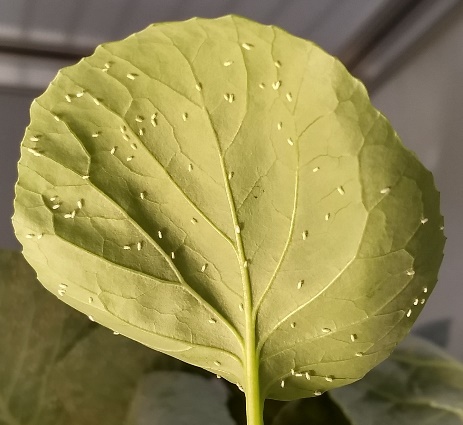


**a**

**b**

**c**

# S4 - Whitefly **Qualitative** Inoculation Protocol

The procedure described here provides for a qualitative colony inoculation which is less time consuming and but provides for a healthy synchronized colony. The method relies more on experience and observation to provide sufficient but not excessive whitefly numbers. The whiteflies are introduced into the new colony by capturing them onto an inoculation plant that is enclosed in an acrylic tube. This procedure does not permit quantification of the number of flies; however, the method provides a high level of containment to prevent whitefly escape from the colony. Notably, the whitefly proliferation model in this paper can be used to back-calculate the number of inoculated flies. It is desirable to avoid transmission of materials from the ‘mature’ cage to keep the whitefly colony clean despite the presence of extensive sugar-containing ‘honeydew”.

Basic procedure to inoculate a new cage with a controllable but **unknown quantity** of flies. Details for fabrication of the transfer tube device are provided at the end of the procedure.

1. **Prepare inoculum Sunday:**
   1. **Select a large healthy 5-week-old cabbage plant** as this will be abused more than the other plants
      1. Measure the weight of the plant **before** watering: if total plant weight is less than 300 g, add up to 30 mL of water.
      2. Option: Take a picture of the plant with a ruler as size reference for leaf surface area image analysis. Ensure the leaf surface is visible.
   2. Place the chosen plant in a PVC/ Acrylic transfer tube for whitefly acquisition.
      1. Wipe down a 6” acrylic tube with the anti-static drier sheet to reduce whiteflies being trapped on static material.
      2. Place a ‘yogurt lid’ in the bottom of the tube, and gently slide the inoculum plant into the acrylic tube, ensuring the tube with the plant inside can be capped. Note that for a longer tube (8-10” tall), using a heavy wire loop is an effective means at placing the pot down into the tube with minimal plant leaf damage (Figure S4-1a).
   3. Capture approximately 100 whiteflies on the inoculum plant in the transfer tube.
      1. Place the transfer tube containing the inoculum plant into the mature cage (at the end of the 6^th^ week, corresponding to two generations of emergence).
      2. The whiteflies will often take ~10 min to move to the plant, be careful to frequently check and keep track of time.
      3. Place cap on the transfer tube when sufficient whiteflies have arrived.
2. **Prepare host plants**:
   1. On every other Monday, set up four fresh cabbage plants in the cleaned and dry cage. Note: a pink 1020 flat bottom flat provides valuable contrast for image analysis. (Figure S4-1b).
3. Introduce the inoculum plant to the new cage; remove the cap and set to the side to remove later (Figure S4-1c).
4. On Tuesday, remove the whitefly transfer tube apparatus.
5. **Iterate the cage numbers**.
   1. The use of stickers to indicate the length of time that the cage has spent in the colony eliminates confusion and ensures a smooth iterative process. Our nomenclature is as follows:
      1. The new cage becomes **Cage 1-2** (having spent 0-2 weeks in the colony), and each subsequent cage is likewise iterated. For example, **Cage 3-4** (having spent 3-4 weeks in the colony) is starting its 5^th^ week and therefore becomes **Cage 5-6.**  Finally, the old **Cage 5-6** is starting its 7^th^ week and therefore becomes **Cage 7-8** which will undergo QC analysis the next day on Tuesday.

**Figure S4-1:** Qualitative whitefly inoculation of a new cage. (a) wire hook facilitating the placement of inoculation plant into the inoculation tube; (b) four other colony plants distributed on a flat with a ruler for surface area measurements; and (c) inoculation plant with the inoculation tube cap removed and placed abreast the other plants – releasing the whiteflies.


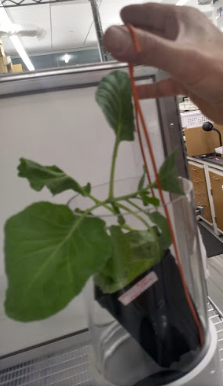

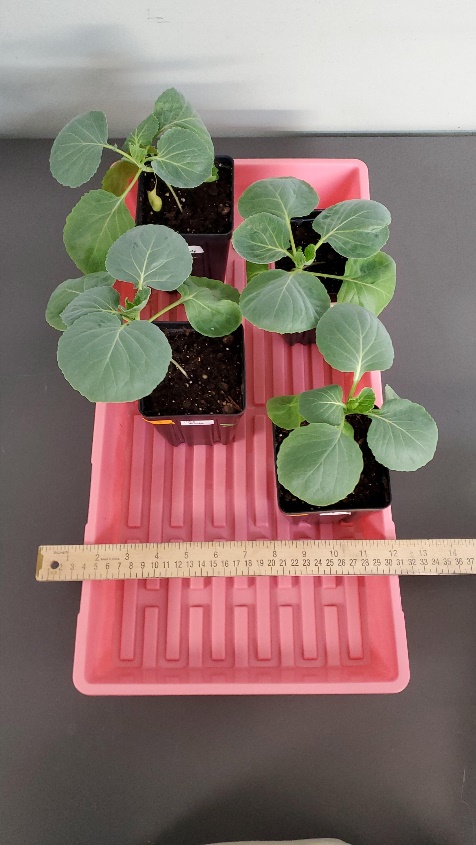


**a**

**b**


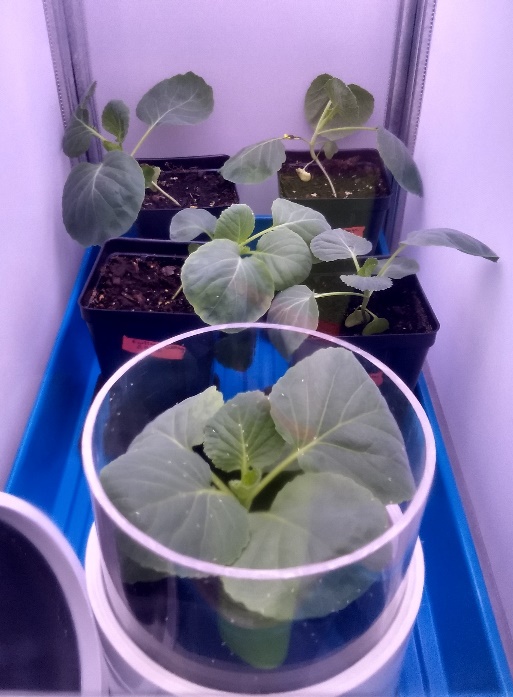


**c**


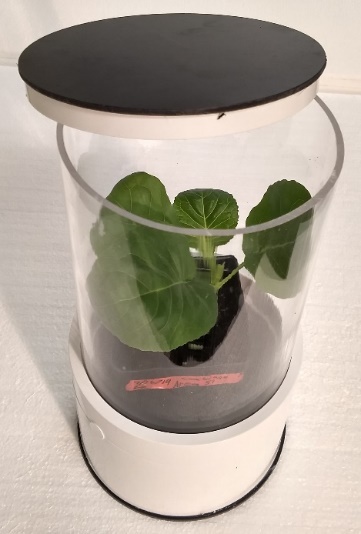
Fabrication of the transfer tube: The transfer tube is fabricated from a PVC pipe coupler (LASKO, 5-in Schedule 40, part #429050) that provides a snug fit to a 5.5-in extruded acrylic tube (Canal plastics, #RT-300128).  A lid was fabricated from a 0.5-inch height ring was cut from the bottom coupler on a lathe and silicone glued to a 1/8-in black ABS plastic (US Plastics, #45353 cut with a 165 mm Milwaukee Tool circular saw).  The bottom coupler was similarly enclosed with a ABS plastic disk, and the upper joint was cut down to 1-in for insertion of the acrylic tube coupling (see Fig. S4-2). The transfer tube allowed for visualization of the whitefly infestation, followed by covering and introduction into the new colony. A configuration with three-spacers cut from 15 mL conical centrifuge tubes to hold the acrylic tube up with a ~1 in gap for whitefly entry can expedite whitefly transfer but is more difficult to handle.

**Figure S4-2:** Tall Whitefly Inoculation Tube

# S5 – Quality Control Procedures

Overview: This describes the procedure of quantitative monitoring of insect colony productivity. The goal is to accurately observe the ‘health’ of the colony from iteration to iteration and to enable troubleshooting of maintenance procedures. There are two weekly quantitative measures: (1) adult flies stuck to sticky paper, and (2) emerged exoskeleton counting.

1 – Whitefly Sticky Count

The amount of healthy flying flies is assessed through a stationary collection method. A 16 cm^2^ yellow (insect attracting) sticky card is placed on a vertical stick in the middle of the cage (next to plant #3) for a minimum of 15 minutes.

Basic procedure to perform a sticky card count on the Tuesday after a cage completes its 6^th^ week.

1. **Prepare Sticky Card:**
   1. **Gather materials**
      1. Vertical metal stick (banquet table number holder works well)
      2. Clean disposable 90mm Petri dish (for an excised leaf)
      3. 2in x 2in yellow sticky card
         1. Cards come in much larger sizes; cut to the appropriate size
   2. Attach yellow sticky card vertically at the top of the metal stick
2. **Open the cage and quickly place the metal stick in the cage and close cage door**
   1. Check Figure S5-1c for placement in the cage – consistency is key.
3. **Check the sticky card after 15 minutes** by taking a quick picture and counting the number of flies in the picture. See Figure 2a for a full picture of a sample card.
   1. If there are more than 15 flies, continue to step 4
   2. If there are fewer than 15 flies, check again in 15 minutes and repeat.
   3. If at 3 hours there are still fewer than 15 flies, remove and count as normal.
4. Once sufficient flies have been reached (or 3 hours has elapsed), put the clean Petri dish into the cage through the cage sock, enclose the sticky card inside the Petri dish, and remove the Petri dish.
5. Count the number of flies on the sticky card and record the time duration it was in the cage.

2 – Emerged Exoskeleton Count

The number of emerged flies and relative fecundity of the colony is assessed through a stationary collection method of a leaf sampling. A uniformly aged leaf is selected

Basic procedure to perform the exoskeleton count on the Tuesday after a cage completes its 6^th^ week.

1. **Select leaf for QC:**
   1. **Choose a consistent aged leaf and plant # for each assessment**
      1. We chose the second full leaf from the base of the 3^rd^ plant in the cage (in the middle). Choosing an older leaf is critical, it must reliably be there for the last month of the 42 days of the colony to ensure consistency.
2. **Slightly mark a circle of 1” diameter into the leaf using a hole punch**
   1. Do not actually sever the leaf using the punch
   2. Choose the area of leaf near the midrib representative of emerged exoskeletons
3. **Count the number of emerged exoskeletons** by taking a picture and manually counting the number of emerged exoskeletons based on the white empty appearance of the nymph shell. Note: we use a stereo microscope for easy counting. See Figure 2b for a zoomed in picture of an emerged exoskeleton.
   1. If there are more than 25 exoskeletons, continue to step 4
   2. If there are fewer than 25 exoskeletons, draw a larger concentric circle around the leaf punch and count again. Repeat until the number of exoskeletons inside the circle exceeds 25.
   3. Calculate the area enclosed in the final circle using the precise 1” diameter of the hole punch impression for calibration
4. Record the number of exoskeletons/in^2^.

**Figure S5-1:** Whitefly Sticky Count: quality control procedure. (a) highly covered whitefly sticky trap showing many flies, (b) sparser sticky trap showing a less prolific colony, and (c) placement of the yellow card inside the cage for consistent quality control sampling. The manuscript text describes the procedure for covering the sticky paper with a plastic bag to allow settling of the colony.


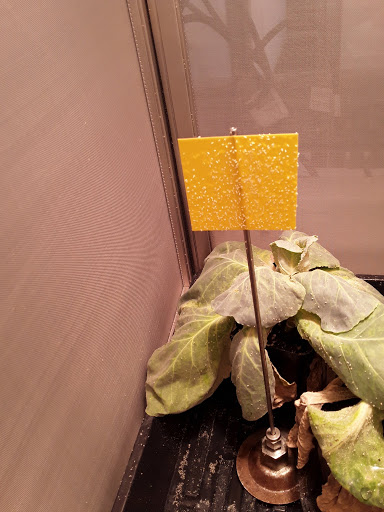

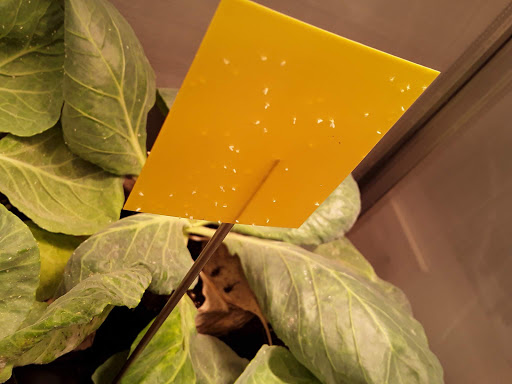


**a**

**b**

**c**

\

**
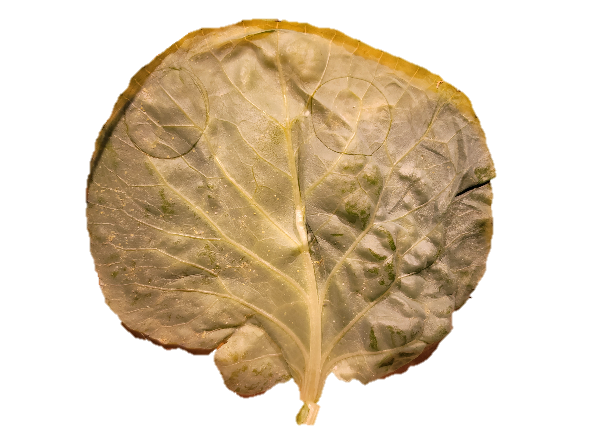
**

**Figure S5-2:** Exoskeleton Count Procedure: quality control on the 2nd fully developed leaf from the bottom of the 3rd plant in the cage. Black circle indicates the drawn concentric circle around the location of the leaf punch. Expansion of the count area as needed to obtain a minimum of 25 exoskeletons.

# S6 – Scaling Transfer from Small Colony

Overview: Although the goal of the colony was scale-down for cost and convenience, our efforts also included approaches to scale back up to use of whiteflies in larger scale studies. This was accomplished both by scaling the cabbage host plant and developing a method to capture large quantities of whiteflies without damaging them. The ‘low Reynolds number whitefly vacuum’ was based on concept of creating a vacuum that had low flow that would then pass through a large surface area filter unit to avoid damaging flies.

Fabrication details: The capture device consisted of a Millipore CRK3 (50 mm x 60 mm DIA) cartridge filter configured into a vacuum device that fits within a wide-mouth mason jar. The concept is to create a vacuum that is sufficiently high flow in the 0.5-inch capture tube (aluminum to avoid static buildup that traps flies), while providing near zero flow at the filter interface to avoid trapping the flies. This was accomplished through vacuum inlet (tapped into the lid of a 50 mL conical centrifuge tube with the bottom cut off and attached to the 25 mm cartridge filter double O-rings). To achieve the fit of the centrifuge tube to the filter, it was necessary to heat the conical centrifuge tube in an autoclave, and then press a second conical tube into the cut off end while it was still hot and somewhat flexible to achieve a secure press fit of the cartridge). Silicone tubing was used for connections both for flexibility and avoidance of whitefly capture.

**Figure S6-1**: whitefly capture device for large numbers of healthy whitefly transfer. **A**) the filter / vacuum design, **B**) the device with hundreds of flies captured without damage, **C**) large format cabbage plants used for scaleup.

Implementation: By attachment to a vacuum pump and throttling flow appropriately, hundreds to thousands of whiteflies could be rapidly captured (Figure S6-1B). The captured flies could then be transferred to a cage containing two larger cabbage plants to rapidly amplify to thousands of whiteflies in less than a month. The pictured ‘plant pot’ is a 6” diameter PVC pipe (penny for reference) and drain with screen in bottom to retain soil. The 18” cube screen cage was full front open, ½ 2x front sock; ½ upper view (Bioquip# 1450NS78). A video demonstrating operation / capture is available on the [www.curtislab.org](http://www.curtislab.org) website.

# S7 – Image Analysis

Overview: Image analysis was used to assess host plant surface area as well as provide automated counting of large numbers of whiteflies.

## 1 – Leaf Surface Area

ImageJ is used for evaluating both the initial surface area and final surface area of the plants entering and leaving the colony. For initial plant leaf surface area, the plants are laid out on a tray with a ruler and a picture is taken from directly overhead as seen in Figure S7-1a. The relative amount of surface area that is not able to be seen due to leaves being curled over or on top of each other is assumed to be consistent from iteration to iteration. This assumption does not need to be made for the whitefly harvest when leaving the colony – the leaves are all individually cut off and laid out on an even surface as seen in Figure S7-1b. These images are then analyzed in this general procedure:

1. **Set Image Scale** – Figure S7-1a&b
   1. Load photo into ImageJ
   2. Using the line tool, draw a line on top of the ruler that is the length of 1 inch.
   3. Calibrate ImageJ by selecting “Set Scale” and setting the length of the line (in pixels) to 1 inch.
2. **Select Exclusively the Leaves** – Figure S7-1c&d
   1. Crop out superfluous surroundings
   2. Change the “Color Threshold” to only select the color green – manipulating saturation and lightness is also helpful depending on the image.
3. **Measure the Surface Area** - Figure S7-1e&f
   1. With the leaves selected, select “Measure” and record the surface area measurement. Note that due to the scale that was set in step 1, the units are in in^2^.

## 2 – Whitefly Cage Harvest Count

Final total whitefly numbers inform and validate both the quality control measures and the model. The following procedure was developed to count thousands of flies when harvested from an old whitefly cage after being brushed onto a flat dark surface.

1. **Perform small-scale calibration** – Figure 3a-b
   1. Crop a small square from the center of the image and count the number of flies by hand
   2. Change the format of the image to 8-bit
   3. Adjust the “Threshold” of the image until only whiteflies are selected and apply the threshold (Figure 3a). Make sure to take note of what the threshold value applied is.
   4. “Analyze Particles” with a size limitation that includes the area of a whitefly and view the outlines (Figure 3b).
   5. Compare the ImageJ count to the hand-count: if they match - continue to step #2, and if they do not match, repeat step d and adjust the size limitation accordingly until the two counts are equivalent.
2. **Analyze Full Image** – Figure 3c-d
   1. Crop out superfluous surroundings and change the format of the image to 8-bit (Figure 3c)
   2. Change the “Threshold” to the exact value used in the small-scale calibration and apply.
   3. “Analyze Particles” with the exact size limitation that was used in the small-scale calibration and view outlines to visually check the accuracy of the count (Figure 3d)


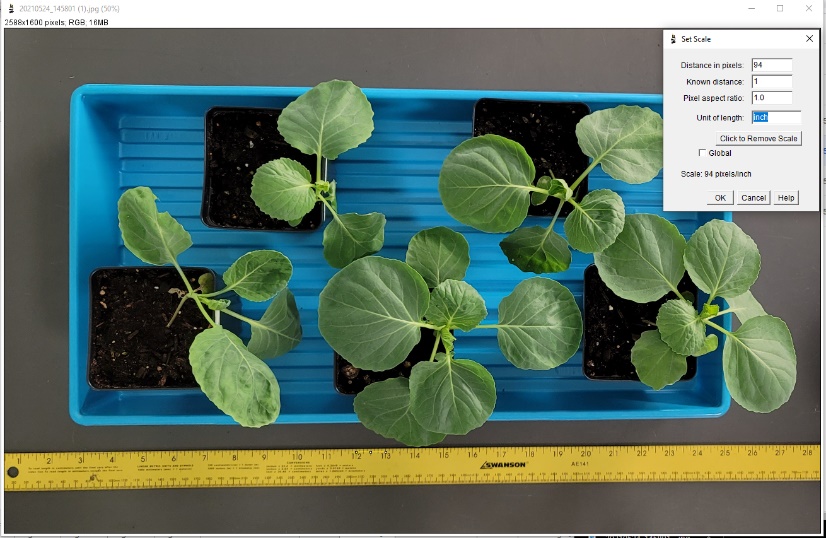

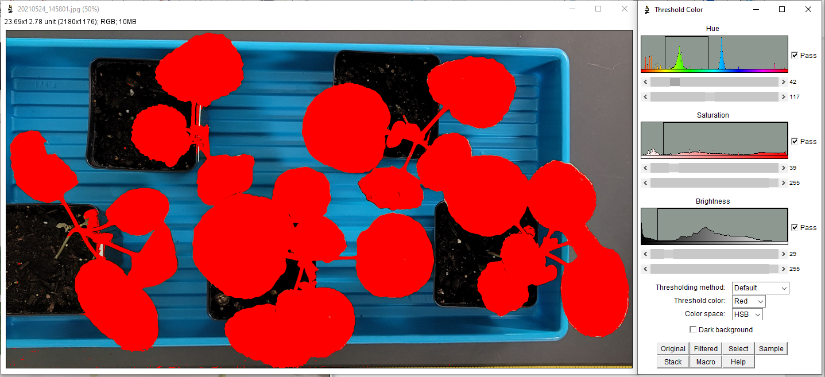

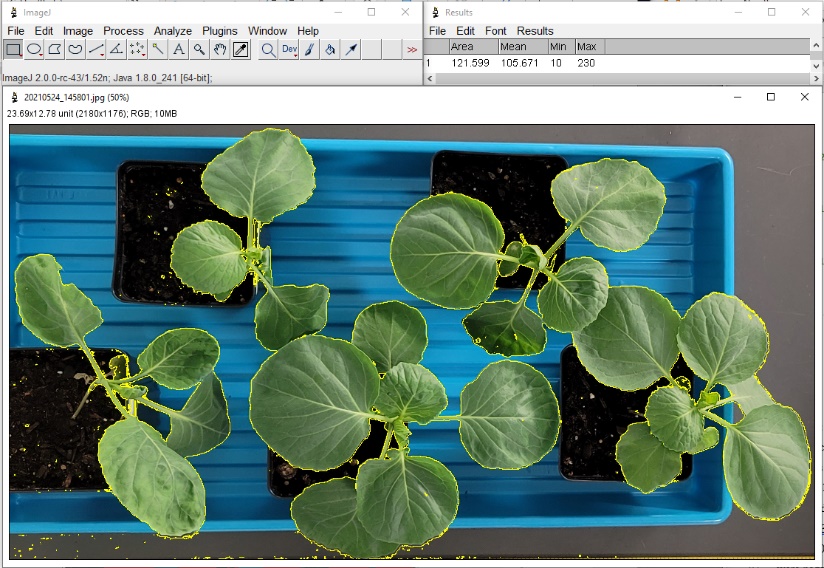

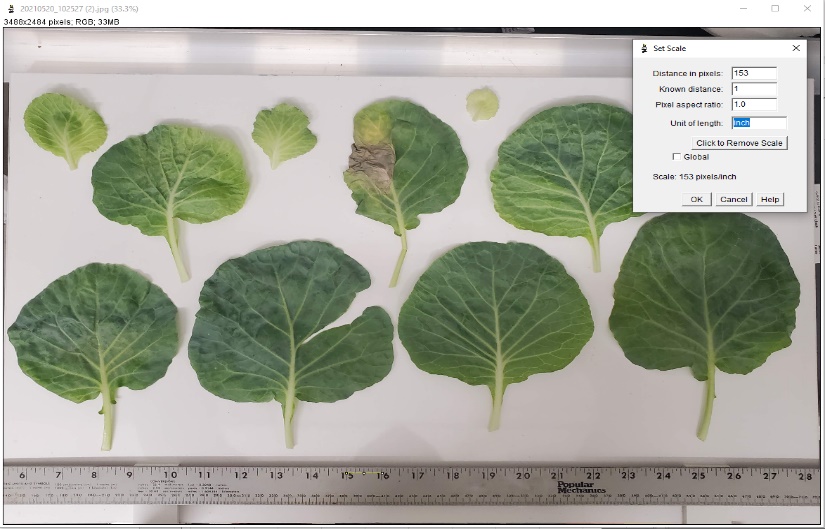

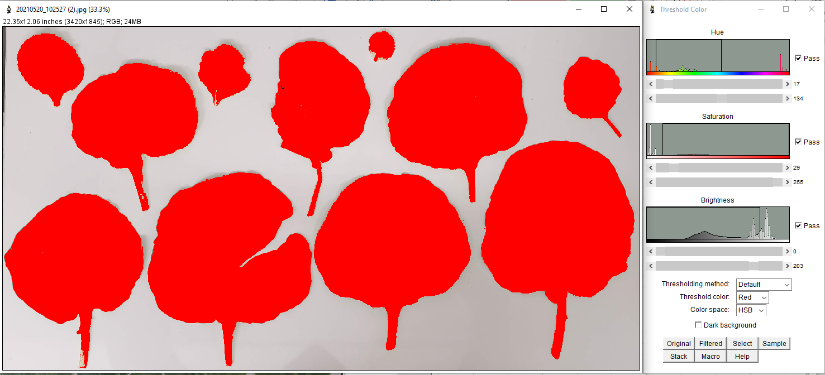

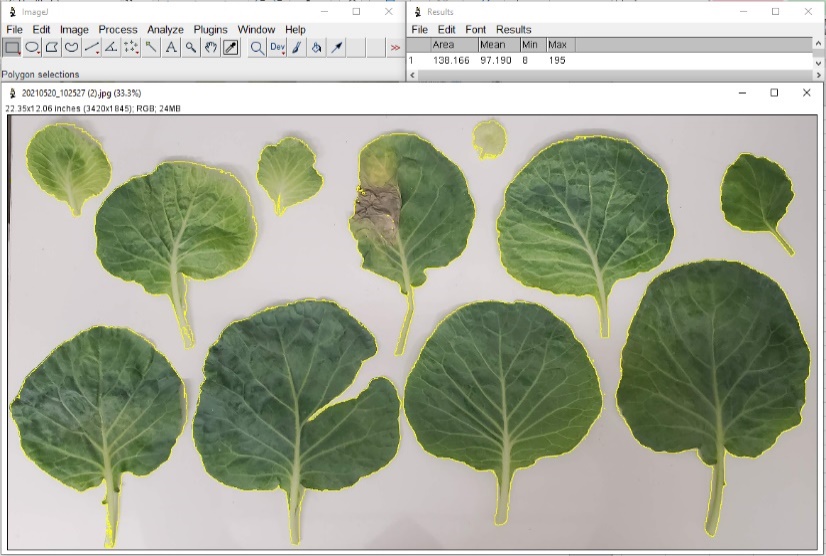


**a**

**b**

**c**

**d**

**e**

**f**

**Figure S7-1:** Image analysis examples of (a,c,e) full cabbage plants when entering the colony and (b,d,f) exiting the colony. The black arrows in (a,b) indicate the inch being selected for scaling image dimensions. The red circles in (e,f) indicate the surface area measurements in in^2^, (e)- 121.599 in^2^ (f) – 136.166 in^2^. Note that analyzing the exit plant size (b,d,f) is done for each plant individually and then the total surface area for each plant is averaged. When analyzing the plants entering the colony, all of the plants are done simultaneously and then the resultant total area is divided by 5.

# S8 – Supporting Images

The following supplemental figures are provided for visual substantiation of observations referenced in the manuscript.


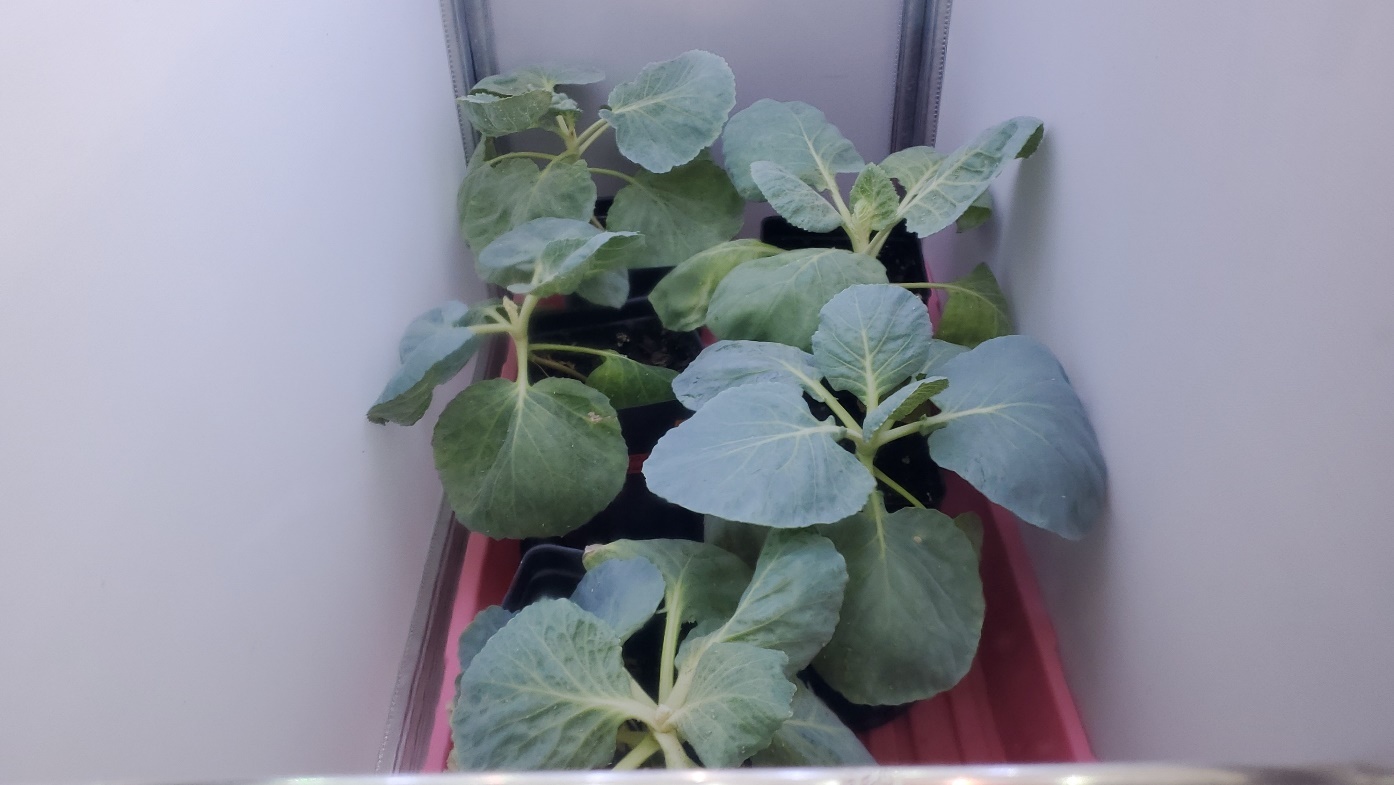

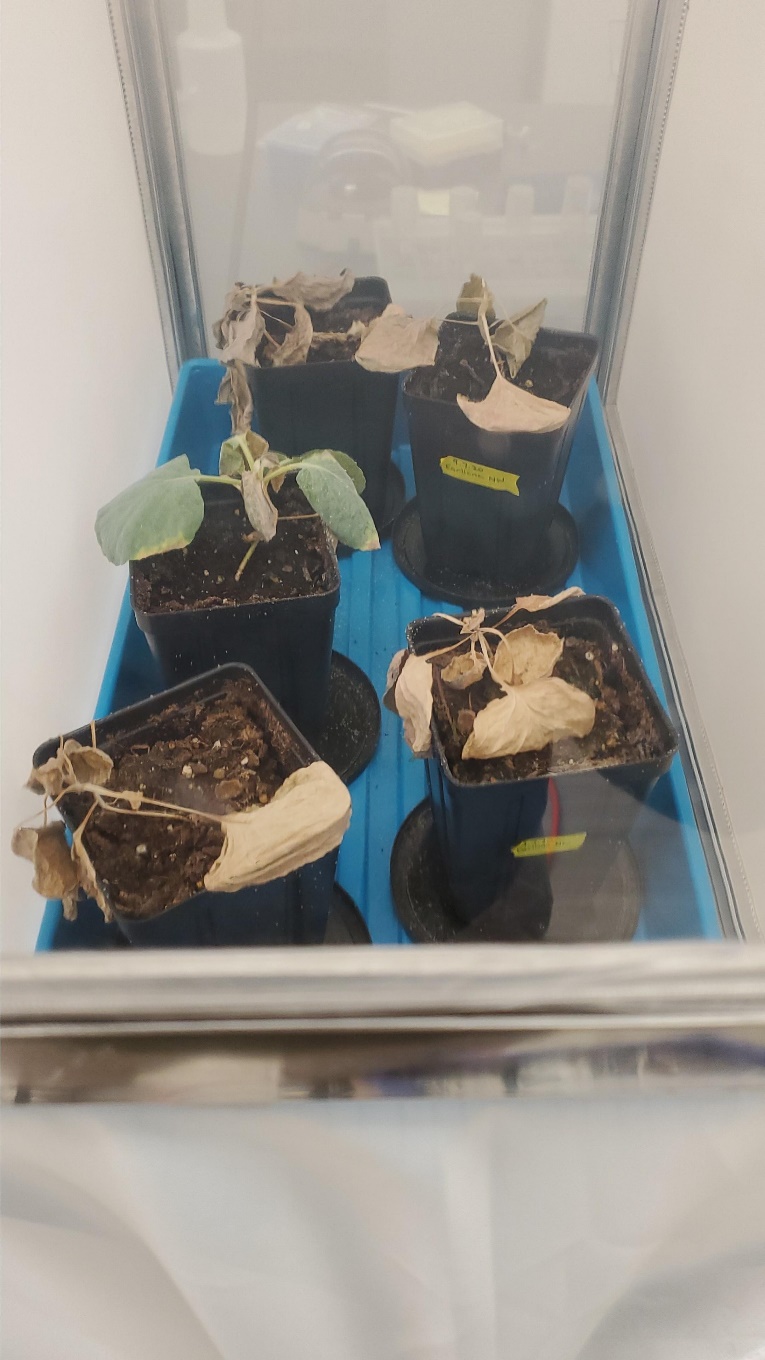


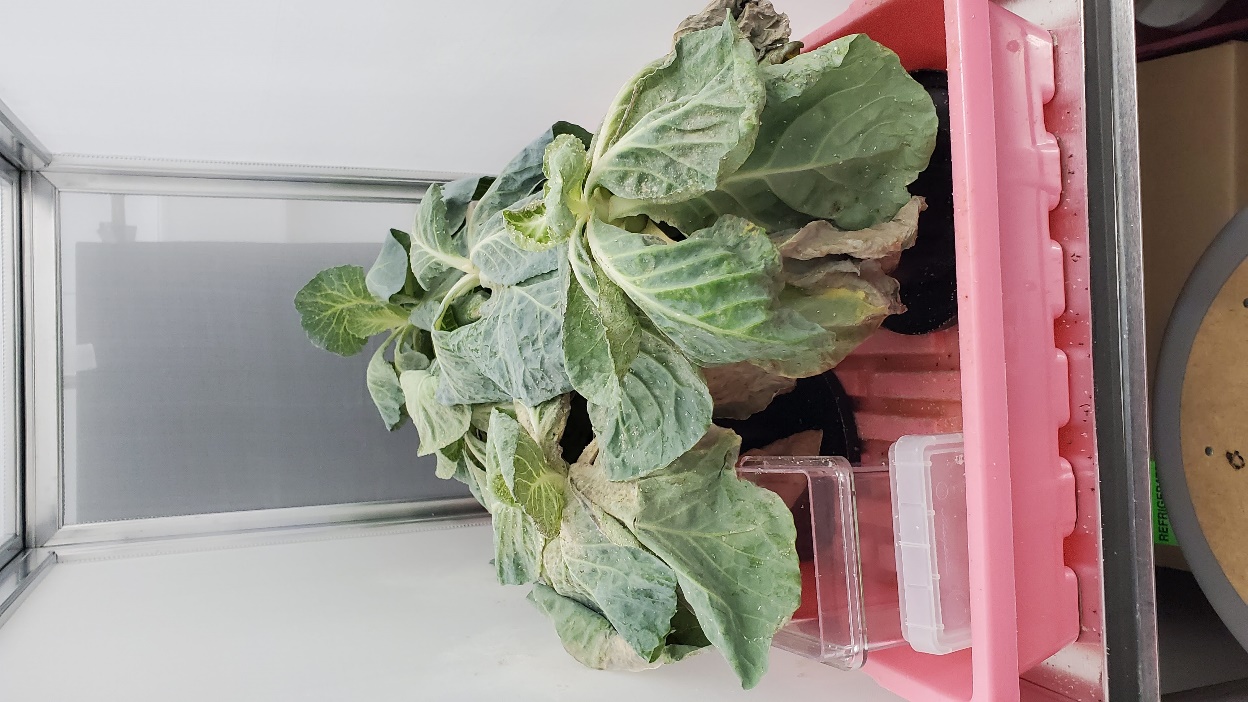


***Figure S8-2****: Picture of a typical cage at harvest with moderate inoculation (Figure 5, iteration 8)*

***Figure S8-3****: Picture of plants from high (~200 whitefly) inoculation at harvest (Figure 5, iteration 13) showing profuse unhealthy grow conditions*

**Figure S8-4**: Inoculation leaf with whiteflies feeding on it. Black circle shows male/female pairs.


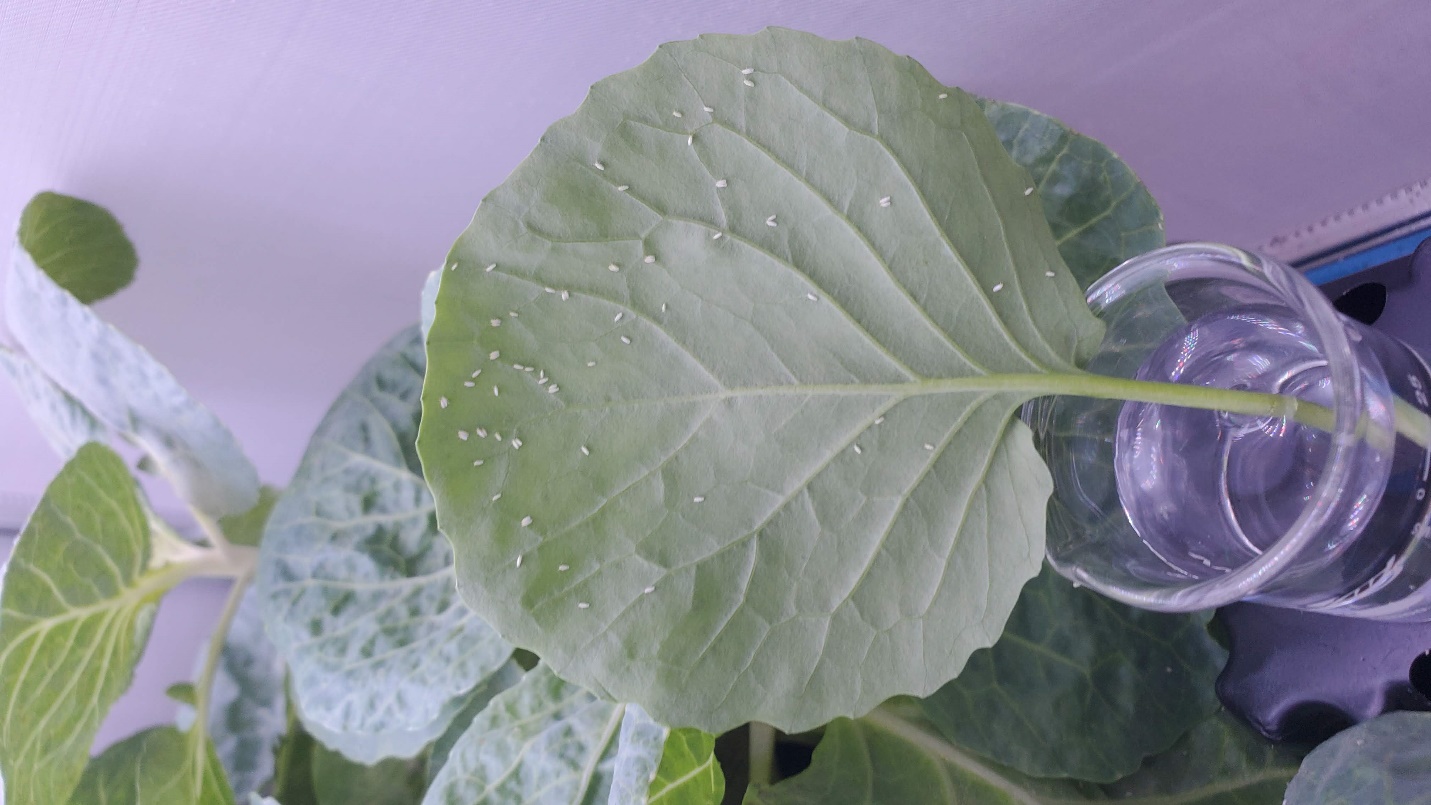

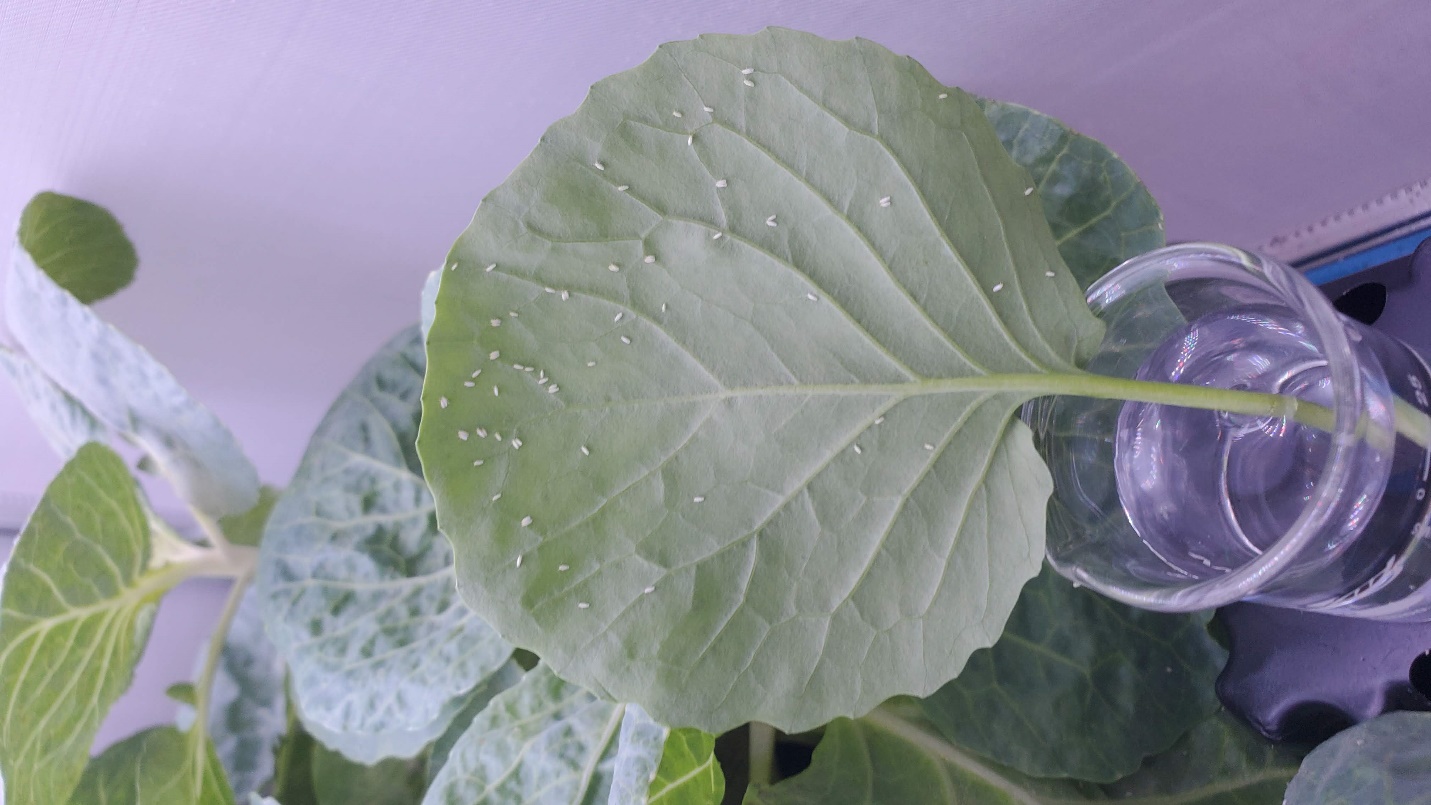


***Figure S8-1****: Picture of colony collapse (dead plants) from early maintenance*
